# Quantitative live-cell imaging of secretion activity reveals dynamic immune responses

**DOI:** 10.1016/j.isci.2024.109840

**Published:** 2024-04-27

**Authors:** Mai Yamagishi, Kaede Miyata, Takashi Kamatani, Hiroki Kabata, Rie Baba, Yumiko Tanaka, Nobutake Suzuki, Masako Matsusaka, Yasutaka Motomura, Tsuyoshi Kiniwa, Satoshi Koga, Keisuke Goda, Osamu Ohara, Takashi Funatsu, Koichi Fukunaga, Kazuyo Moro, Sotaro Uemura, Yoshitaka Shirasaki

**Affiliations:** 1 Graduate School of Pharmaceutical Sciences, The University of Tokyo, Tokyo 113-0033, Japan; 2 Department of Biological Sciences, Graduate School of Science, The University of Tokyo, Tokyo 113-0033, Japan; 3 Live Cell Diagnosis, Ltd., Saitama 351-0022, Japan; 4 Division of Pulmonary Medicine, Department of Medicine, Keio University School of Medicine, Tokyo 160-8582, Japan; 5 Department of AI Technology Development, M&D Data Science Center, Tokyo Medical and Dental University, Tokyo 113-8519, Japan; 6 Division of Precision Cancer Medicine, Tokyo Medical and Dental University, Tokyo 113-8519, Japan; 7 Department of Microbiology and Immunology, Graduate School of Medicine, Osaka University, Suita, Osaka 565-0871, Japan; 8 RIKEN Center for Integrative Medical Sciences, Yokohama, Kanagawa 230-0045, Japan; 9 Department of Chemistry, Graduate School of Science, The University of Tokyo, Tokyo 113-0033, Japan; 10 Department of Bioengineering, University of California, Los Angeles, Los Angeles, CA 90095, USA; 11 Institute of Technological Sciences, Wuhan University, Hubei 430072, China; 12 KAZUSA DNA Research Institute, Kisarazu, Chiba 292-0818, Japan

**Keywords:** Optical imaging, Immune response, Transcriptomics

## Abstract

Quantification of cytokine secretion has facilitated advances in the field of immunology, yet the dynamic and varied secretion profiles of individual cells, particularly those obtained from limited human samples, remain obscure. Herein, we introduce a technology for quantitative live-cell imaging of secretion activity (qLCI-S) that enables high-throughput and dual-color monitoring of secretion activity at the single-cell level over several days, followed by transcriptome analysis of individual cells based on their phenotype. The efficacy of qLCI-S was demonstrated by visualizing the characteristic temporal pattern of cytokine secretion of group 2 innate lymphoid cells, which constitute less than 0.01% of human peripheral blood mononuclear cells, and by revealing minor subpopulations with enhanced cytokine production. The underlying mechanism of this feature was linked to the gene expression of stimuli receptors. This technology paves the way for exploring gene expression signatures linked to the spatiotemporal dynamic nature of various secretory functions.

## Highlights

• We introduce quantitative live-cell imaging of secretion activity (qLCI-S)
• It enables long-term and dual-color quantitative tracking of cell secretion activity
• qLCI-S is integrated with transcriptomic analysis of identified phenotypic cells
• qLCI-S elucidates minor yet effective subpopulations within immune cells

## Graphical abstract

**Figure:**
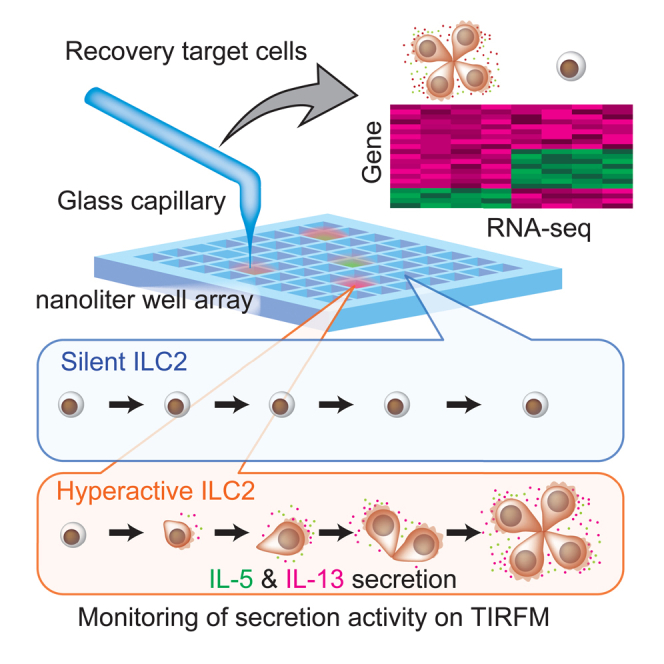

## Introduction

Secretion is a crucial cellular function that facilitates intercellular communication via soluble messengers such as cytokines, hormones, neurotransmitters, growth factors, and extracellular vesicles.^1^^,^^2^^,^^3^ Perturbations in the cytokine network are believed to play a role in a wide range of pathological conditions, including infection, chronic inflammation, allergies, aging, cancer progression, and autoimmune diseases.^4^^,^^5^^,^^6^^,^^7^^,^^8^ Consequently, the regulation of cytokine secretion is considered finely tuned in various biological systems, including the immune system.

Group 2 innate lymphoid cells (ILC2s) are unique lymphocytes with the ability to produce large amounts of cytokine.^9^^,^^10^ They are believed to significantly contribute to the chronic allergic diseases such as asthma^10^^,^^11^ because such diseases are not preciously explained by Th2 cells, which are traditionally regarded as the primary producers of type 2 cytokines. Unlike conventional lymphocytes, ILC2s do not express antigen receptors but are activated through cytokine receptors. For instance, interleukin-2 (IL-2) promotes moderate proliferation of ILC2s. Additionally, IL-2 enhances type 2 response of ILC2s, such as the production of IL-5 and IL-13, in response to IL-25, a cytokine produced by tuft cells.^12^ Furthermore, the alarm cytokine IL-33^13^ accelerates the secretion of type 2 cytokines along with the rapid proliferation of ILC2s. A major challenge in the characterization of ILC2s is the limited number of cells that can be isolated from human specimens. Therefore, for functional assessment of human ILC2s, it is necessary to expand the number of isolated ILC2s by culture,^14^ but the impact of this culture on ILC2 properties is not yet understood.

To address this issue, techniques that detect and track cytokine secretion at the single-cell level offer a solution.^15^ Among them, live-cell imaging of secretion activity (LCI-S), a time-resolved fluorescence immunospot assay performed using total internal reflection fluorescence (TIRF) microscopy (TIRFM), has successfully observed the release of alarmins associated with cell death.^16^^,^^17^^,^^18^^,^^19^ However, it is difficult to quantitatively reveal the dynamic secretion activity of living cells using this technique, due to inability of both the optical setup and the culture environment to sustain stability for more than one day.^20^^,^^21^ At present, this technique has solely been utilized to quantitatively analyze the release rate and the timing of secretion onset, without addressing long-term stability measures. In this study, we report a quantitative LCI-S platform for long-term tracking of the cytokine secretion activities in multiple colors in individual cells. The combination of a cell-harvesting tool with the platform facilitated gene expression analysis of targeted cells identified by their secretion activities.

## Results

### Establishment of LCI-S quantitative analysis

**Figure 1:**
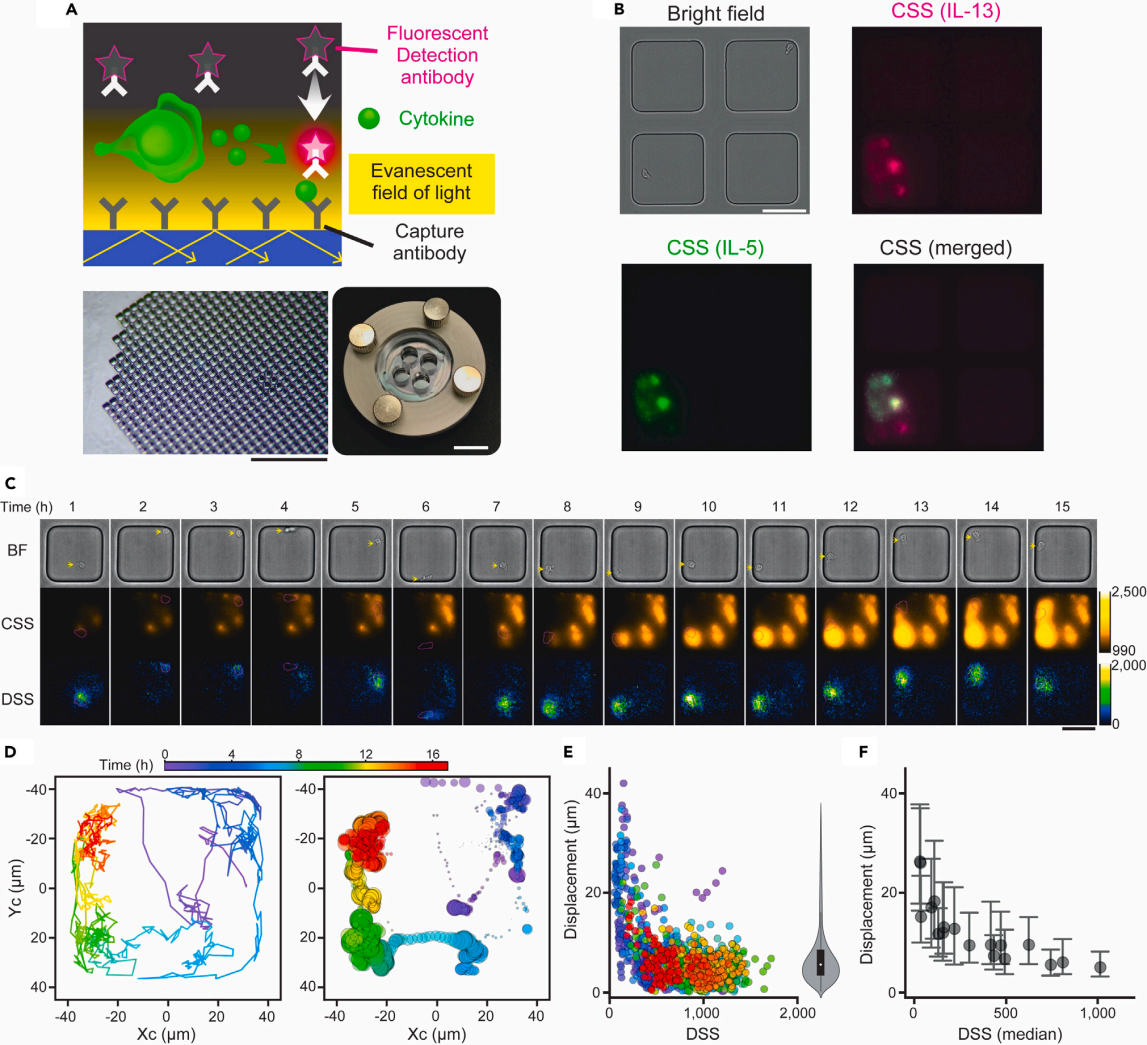
Establishment of qLCI-S for spatiotemporal secretion analysis (A) Schematic of qLCI-S measurement principle (upper) and overview of the TIRF dish (lower, right). The TIRF dish consists of a nanoliter well array with 996 wells fabricated as cubes with 80 μm on a side (lower, left) on the bottom surface of each of the four chambers. Scale bars: 1 mm (lower, left) and 10 mm (lower, right). (B) Representative example of qLCI-S data obtained from a single field of view. Bright-field (left), IL-13 signal (magenta), IL-5 signal (green), and IL-5/IL-13 superimposed images (merged) are shown. Scale bar: 50 μm. (C–E) Examples of spatiotemporal deconvolution strategies for qLCI-S signals, utilizing data sourced from the well located at the bottom left in Figure 1B. (C) Bright-field images of a mouse innate lymphoid cell (mILC2; top, BF), cumulative secretion signal (CSS) images of IL-5 (middle, CSS), and temporally deconvoluted secretion signal (DSS) images (bottom, DSS). Images at representative points in time from the data acquired and analyzed every minute are shown. Scale bar: 50 μm. See also Video S1. (D) Left: The movement of the geometric center of ILC2 (Xc, Yc) at each time point is shown as a trajectory drawn in time-coded colors. Right: The 2D Gaussian distributions fitted to the DSS signal at each time point are indicated by the position (Xc, Yc), peak height (1 μm of bin size), and color (time) of the circles. (E) Relationship between the intensity of the DSS and the distance of its center from the cell; the higher the intensity, the closer the central position of the DSS to the cell location. The right violin plot shows the distribution of the distances with a median of 5.6 μm. (F) Relationship between the median intensity of the DSS and the median distance of its center from the cell obtained from 18 individual cells. Error bars indicate the 1st quartile and 3rd quartile of the distance.

We devised quantitative live-cell imaging for secretion activity (qLCI-S), to continuously monitor the secretion activity of living single cells over several days. qLCI-S was performed on a unique glass/resin hybrid culture dish that featured a microfabricated array of nanoliter wells and leveraged a time-resolved fluorescence immunoassay on the bottom surface of each well, visualized through TIRFM (Figure 1A). To address the inherent optical instability, TIRF illumination incorporating light-emitting diode (LED) light sources was implemented to reduce spatiotemporal noise (Figure S1). This development was aimed at improving detection accuracy and enabling extended observation periods by achieving uniformity and stability. In this study, we utilized a qLCI-S platform with a TIRF dish consisting of four chambers, each containing 996 wells at the base (Figure 1A).

The efficacy of the qLCI-S method was assessed utilizing ILC2s that were expanded *in vitro* from mouse adipose tissue (mILC2), renowned for its pronounced secretion activity.^9^ Figure 1B depicts a snapshot of monitoring IL-5 and IL-13 secretion from mILC2 using qLCI-S during the process. In the top-right well, a single mILC2 was introduced, and one was introduced in the bottom-left well; however, only the bottom-left well exhibited localized secretion signals at its base. Notably, the secretion signals depicted here embody the cumulation of all secretion activity that occurred from the time the cells were introduced into this system until the present moment, hereinafter referred to as the cumulative secretion signal (CSS). It should be noted that there is concern that isolation of individual cells within the nanoliter space may affect their activity, and indeed semi-enclosed cultures were suitable for mILC2s (Figure S2).

Next, we assessed the validity of our innovative analytical strategy, which enables temporal deconvolution of a series of secretion signal images obtained through qLCI-S into true secretion kinetics and antibody staining kinetics to extract dynamic secretion activity in a spatiotemporally resolved manner. We computed deconvoluted secretion signals (DSSs) from the CSS image of IL-5 using the method described in the STAR Methods, which employed reference data for the staining kinetics of the IL-5 antibody against the recombinant IL-5 protein under the same measurement conditions (Figure 1C, Video S1). The computed DSS images displayed spatially localized signals. Changes in DSS distribution were observed to be synchronized with cellular movement (Figure 1D). We examined the spatial correlation between DSS and cells to determine if the DSS results were temporally misaligned. Notably, this analysis holds merit only when the DSS distribution is distinctly observable. In cases of low DSS levels, the accuracy of pinpointing the DSS location diminishes, and verification becomes unattainable when DSS is undetectable. As a parameter for the spatial correlation between DSS and cells, we calculated the displacement between the centroid of DSS and the center of cells identified in the bright-field image. When the DSS signals were sufficiently high, the displacements were typically within 10 μm from the cell center (median distance 5.6 μm), indicating DSS signals were localized around cells (Figure 1E). The equivalent results were obtained for 18 cells (Figure 1F). This effectively demonstrated that qLCI-S could be used to visualize dynamic cellular secretion activity with a high spatiotemporal resolution.

### Statistical comparison of secretion activity using the qLCI-S platform

**Figure 2:**
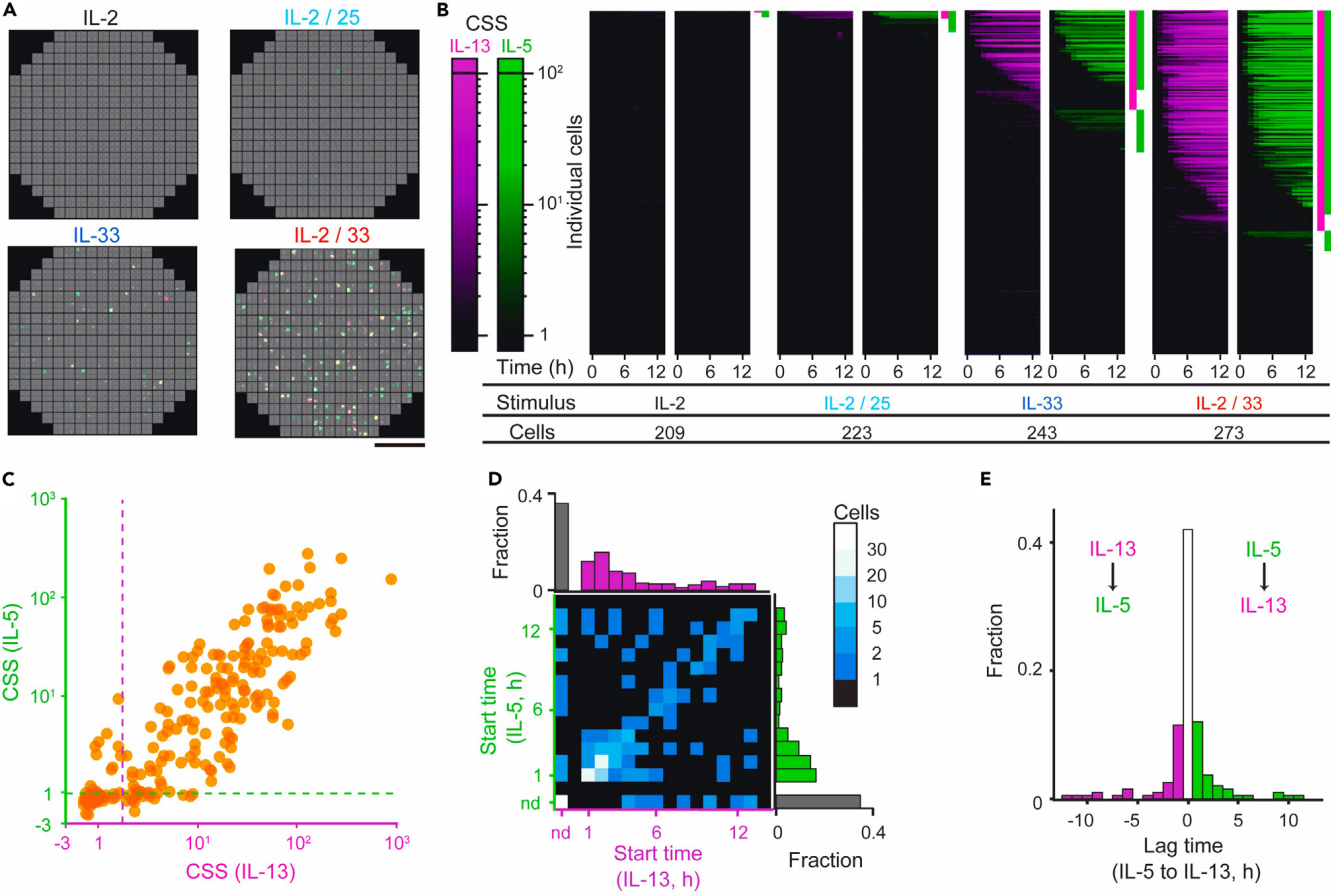
Statistical comparison of secretion activity using the qLCI-S platform (A) Responses from individual mILC2s stimulated with different stimuli (IL-13: magenta, IL-5: green). Each of the 249 fields of view captured four nanoliter wells and was arranged according to its position on the TIRF dish. Scale bar: 1,000 μm. See also Video S2. (B) The chronological heatmap of CSS from the individual mILC2s under different stimuli. Each row reflects the dynamic activity of individual mILC2s for the total number of single cells indicated for each stimulus, whereas the column indicates the time series. The color scale bars show the intensity of CSS (IL-13: magenta, IL-5: green). Time-lapse data were obtained every 1 h. (C) Comparison of the CSS intensity of the two cytokines in each mILC2. Each axis corresponds to a cytokine species, while each point corresponds to a cell. Each CSS is plotted using axis with a separation occurring at CSS (IL-5) = 1 or CSS (IL-13) = 1. The data were plotted on the linear axis below the break and on the logarithmic axis above the break. The dashed line on each axis indicates the detection limit. Spearman rank order correlation coefficient *r* = 0.9, *p* < 0.05. (D and E) Comparison at the start of secretion. (D) Correlation of the time from stimulation to the start of secretion between cytokines; cells without detectable secretion after 12 h of observation were assigned to the origin of each axis in black color as not detectable (n.d.). The numbers of cells in each bin are displayed using a heatmap according to the color bar shown on the right. (E) Differences at the start of secretion between the two cytokines in each cell; 40% of the cells started to secrete simultaneously, but some initially secreted only IL-13 (magenta) and some by IL-5 (green).

Next, we examined how qLCI-S could contribute to comparative studies using the example of stimulus-dependent cytokine production responses of mILC2 to four stimuli known to elicit different responses: IL-2, IL-2/IL-25, IL-33, and IL-2/IL-33 (Figure 2A, Video S2). Prior to the comparative study, we first surveyed the characteristics of IL-5 and IL-13 secretion from individual ILC2. Most of the mILC2s activated with IL-2/L-33 stimulation produced IL-5 and/or IL-13 12 h after stimulation (rate: 0.67). The rate for double-positive cells was 0.57, and the rates for IL-5 and IL-13 single-positive cells were 0.06 and 0.04, respectively (Figure 2B). The intensities of the secreted IL-5 and IL-13 were correlated on a logarithmic scale within cells (Spearman rank order correlation coefficient *r* = 0.9, *p* < 0.05) and substantially differed by more than two orders of magnitude between cells (Figure 2C). Although most active mILC2s (rate: approximately 0.7) started secretion within 5 h of stimulation, the starting points varied widely from 1 to 12 h after stimulation for IL-2/IL-33 (Figure 2D). While nearly all active cells secreted both types of cytokines, the start time of secretion of these was not always coincident (Figure 2E).

Video S2. High-throughput measurements of IL-5 and IL-13 secretion activity of mILC2s, related to Figure 2 Bright-field image and cumulative secretory signal images of IL-5 and IL-13 of mouse group 2 innate lymphoid cell (mILC2) in all wells of TIRF dish were tiled. The intensities of the secretion signals of IL-5 and IL-13 are shown as green and magenta pseudo-colors, respectively. The interval of each image is 1 h. The stimuli added to each chamber are indicated in the top corner of each tiled area.

**Figure 3:**
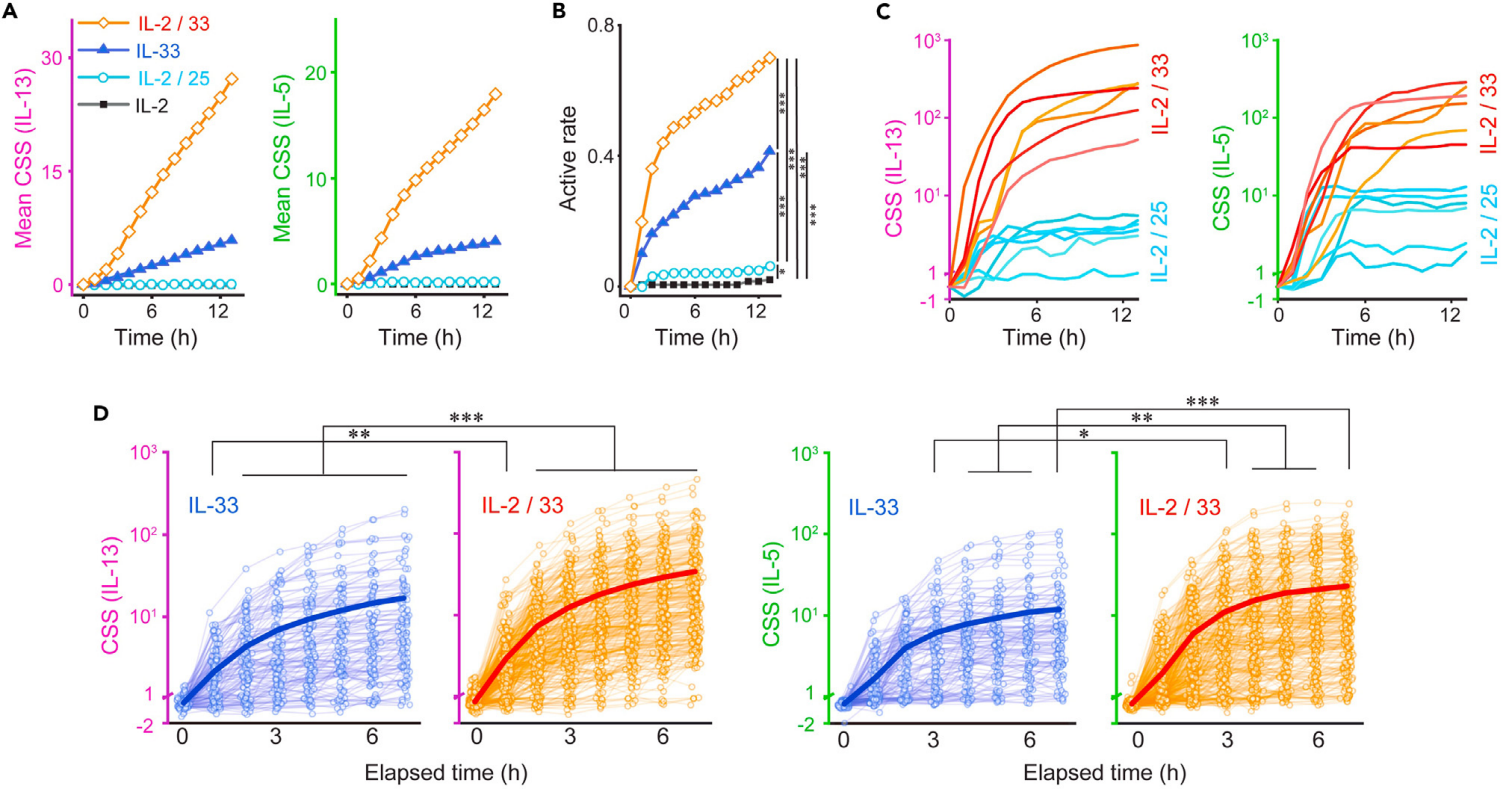
Statistical comparison of time-series dynamics in secretion using the qLCI-S platform (A) Comparison of the mean CSS of ILC2s under different stimuli. The mean CSS was calculated from the total sum of the CSS divided by the total number of analyzed single ILC2s, as comparable to the bulk secretion assay. (B) Comparison of the rate of activation of the ILC2s under different stimuli. ILC2s with a CSS of IL-5 or IL-13 over each threshold based on the CSS of empty wells are shown. Significant differences were assessed for all group combinations using the Benjamini-Hochberg corrected log rank multiple test (∗∗∗*p* < 0.001, ∗*p* < 0.05 with q < 0.01). (C) Comparison of secretion activity between IL-2/25 (blue) and IL-2/33 (orange) stimuli: the traces of the top six in CSS with each stimulus are shown for IL-13 and IL-5 secretion. Each CSS is plotted using axis with a separation occurring at CSS (IL-5) = 1 or CSS (IL-13) = 1. The data were plotted on the linear axis below the break and on the logarithmic axis above the break. (D) Comparison of the secretion activity of individual cells stimulated with IL-33 in the absence (blue) or presence (orange) of IL-2. The evolution of the signal from the start of secretion is aligned. The thin lines show the trace of each cell, and the thick lines show their mean. Differences were assessed using the Mann-Whitney U test (∗∗∗*p* < 0.001, ∗∗*p* < 0.01, ∗*p* < 0.05).

We then performed a comparative analysis among different stimuli. We obtained preliminary confirmation that the secretion activity of mILC2s remained robust regardless of the observation frequency and that the four chambers were quantitatively comparable (Figures S2 and S3). First, we compared the ensemble averages of the secretion signals, which mimic the conventional bulk measurements of the cell population, to determine whether the results obtained align with previous findings (Figure 3A). mILC2 responded most strongly to IL-2/IL-33 stimulation, followed by weaker responses to IL-33, IL-2/IL-25, and IL-2, which agreed with previous studies.^9^ However, significant variability between batches requires multiple repeated and validated measurements to ensure accurate quantitative analysis of ensemble averages. Within batches, this approach allows statistical comparison of secretion activities at the single-cell level, allowing us to detect differences in response to each stimulus (Figure 3B; IL-2 vs. IL-2/IL-25: *p* < 0.05, other combinations: *p* < 0.001), which were comparable to the tendency of ensemble mean of the secretion signals described earlier. Next, our focus shifted toward examining variations in the secretion levels of individual cells in response to distinct stimuli. Comparison of traces of CSS from cells exhibiting high secretion under IL-2/IL-25 or IL-2/IL-33 stimulation showed distinct patterns: Specifically, stimulation with the former resulted in a small amount of transient secretion, whereas stimulation with the latter resulted in a large amount of continuous secretion (Figure 3C). Thus, it is evident that disparities in stimuli influence not only the frequency of secretion cell but also the intensity of the secretion activity. Our subsequent investigation focused on assessing how the simultaneous presence of IL-2 during IL-33 stimulation influenced secretion activity. Although IL-2 alone could not induce cytokine production (Figures 2A, 2B, 3A, and 3B), its concurrent administration with IL-33 stimulation amplified the proportion of cells displaying secretion (as depicted in Figures 2B and 3A). For evaluating the intensity of secretion from individual cells, a non-parametric comparison of the CSS at specified time points following the initiation of secretion revealed a notable rise in CSS when IL-2 was co-administered during IL-33 stimulation, signifying an enhancement in the intensity of the secretion activity of individual mILC2s (Figure 3D; *p* < 0.001). These results demonstrated that the qLCI-S platform provides a high-throughput measurement that allows for statistical comparison of the detailed differences in secretion activity in a small number of cells of the same batch.

### Minor hyperactive ILC2s responsible for secretory response of the population

We utilized the qLCI-S methodology to examine the progression of human peripheral blood-derived ILC2s (hILC2s) during expansion cultivation. It is commonly acknowledged that the fraction of hILC2s is small and inadequate for a thorough analysis of cytokine secretion; conventionally, such assessments necessitate the expansion cultivation of hILC2s prior to analysis.^14^ However, the impact of this cultivation on the *ex vivo* hILC2 functions remains unclear. Consequently, we projected that monitoring the secretion activities of freshly isolated hILC2 (as depicted in Figure S4) via the qLCI-S platform would elucidate the influence of the growth culture process on secretory kinetics, either at the singular cellular level or across the cell population.

**Figure 4:**
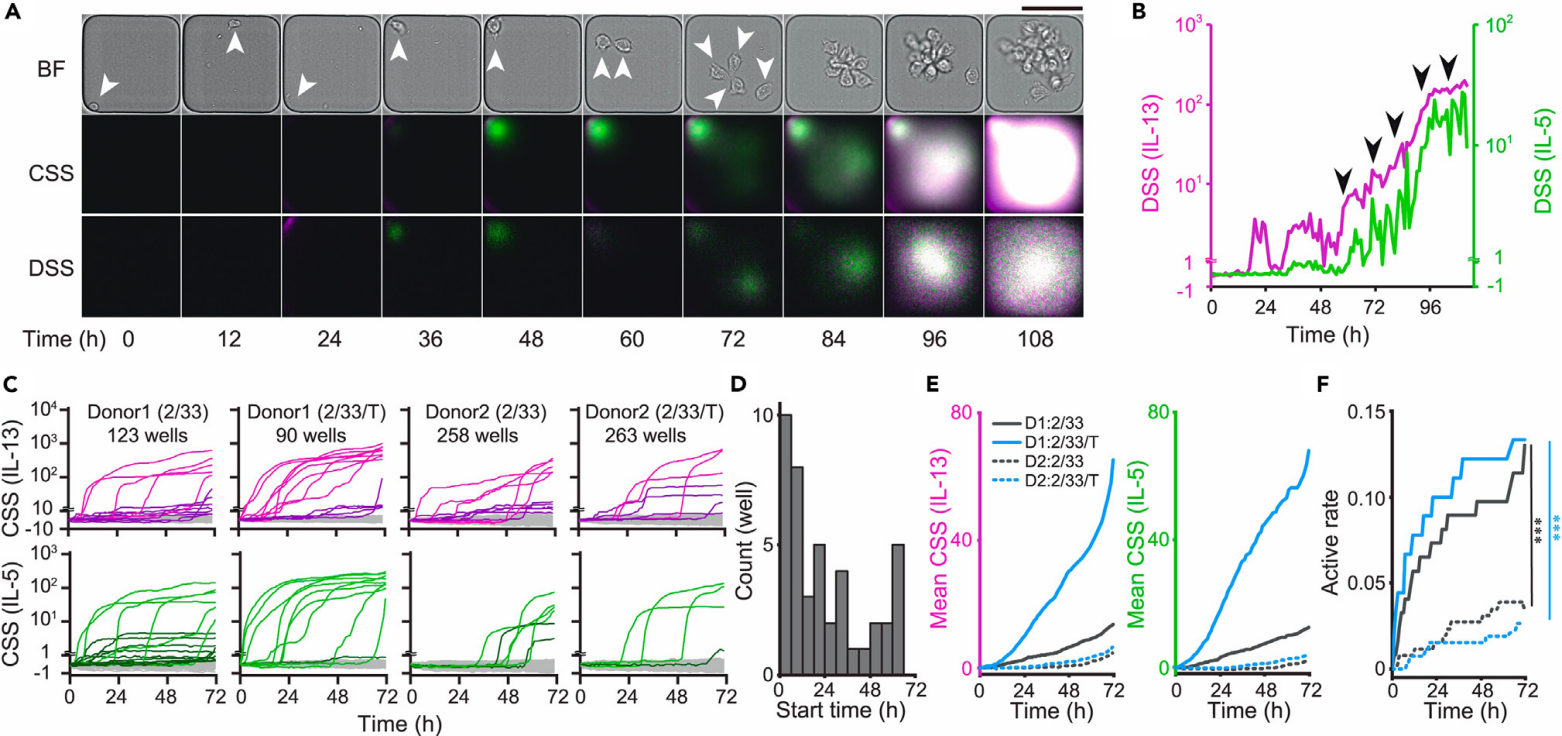
Time evolution of secretory kinetics exhibited by *ex vivo* human ILC2 (A and B) Tracking of hILC2 secretion activity using the qLCI-S platform. (A) Bright-field images of an hILC2 (top, BF); CSS and DSS images in the superimposed color of IL-5 (green) and IL-13 (magenta). Data are shown for every 12 h. Arrowheads indicate the position of hILC2 until the second division. Scale bar: 50 μm. See also Video S3. (B) Temporal changes in the each DSS calculated from the CSS within the well shown in Figure (A). Each DSS is plotted using axis with a separation occurring at DSS (IL-5) = 1 or DSS (IL-13) = 1. The data were plotted on the linear axis below the break and on the logarithmic axis above the break. Arrows indicate the timing when the cell underwent division. (C) Chronological trace of the secretion activity of hILC2s for 3 days. CSS of each well summarized for each donor (D1: donor 1 or D2: donor 2) and each stimulus (2/33: IL-2 and IL-33 stimulation, 2/33/T: IL-2, IL-33, and TSLP stimulation). Each CSS is plotted using axis with a separation occurring at CSS (IL-5) = 1 or CSS (IL-13) = 1. The data were plotted on the linear axis below the break and on the logarithmic axis above the break. Each line reflects individual wells containing a single cell or several cells, as estimated by the AI-supported cell counter. The total number of cell-containing wells is shown for each sample index. Bright, dark, and light-gray lines indicate hyperactive, slightly active, and silent ILC2s, respectively. (D) Distribution of the start of secretion in individual hILC2s. The start of secretion for all cells detected in (C) was made into a histogram, regardless of the stimulus type or donors. (E and F) Comparison of the secretion activity between the two donors with or without TSLP. Comparison between donors and stimulations (solid lines: donor 1, dashed lines: donor 2, black: IL-2/IL-33 stimulation, blue: IL-2/IL-33/TSLP stimulation). (E) Comparison of the mean CSS (IL-5: green, left; IL-13: magenta, right). (F) Comparison of the rate of activation of ILC2s. Differences were tested using a Benjamini-Hochberg corrected log rank multiple test (∗∗∗*p* < 0.001 with q < 0.01).

Fluorescence-activated cell sorting (FACS)-purified hILC2s from human peripheral blood were introduced into TIRF dishes along with stimuli and observed for five days to ensure capturing of the full cellular response (Figure 4A, Video S3). We first focused on a population of hILC2s, which secrete large amounts of IL-5 and IL-13 when co-stimulated with IL-2 and IL-33 and investigated their dynamics. As secretion activity progressed, hILC2s transformed from a small spherical shape to an enlarged shape with a tail and fin-like structures. In most cases, IL-5 and IL-13 secretion activities were observed to be synchronized, though the intensity fluctuated in cycles of around 20 h (Figure 4B). The continuously secreting ILC2s underwent cyclic proliferation and were repeatedly associated and dissociated into a single aggregate. In certain wells, where cells exhibited repetitive proliferation and substantial cytokine production, proliferating cells formed three-dimensional aggregates, rendering cell counting challenging. In these instances, the increase in CSS tended to be stagnant after day 4, raising concerns about potential impacts on capture antibody capacity or accessibility (Figure 4B). Based on this, although the observation itself would continue for 5 days, the analysis of the secretion activity of hILC2 according to the intensity of CSS was performed on data until the third day after stimulation, when the times of divisions exhibited by activated hILC2 were less than three.

Video S3. Visualization of dynamic IL-5/IL-13 secretion activity of hILC2, related to Figure 4 Bright-field images of group 2 innate lymphoid cells from human peripheral blood (hILC2s; upper), cumulative secretion signal (CSS) images of IL-5 and IL-13 (bottom), and temporally deconvoluted secretion signal (DSS) images (middle). The intensities of the secretion signals of IL-5 and IL-13 are shown as green and magenta pseudo-colors, respectively. The interval of each image is 1 h.

The experimental setup for hILC2 was designed with the possibility in mind that a specific feature of hILC2, identified by qLCI-S, might account for individual variations among donors. We statistically compared the secretion activity of hILC2 from different donors stimulated by IL-2/IL-33 with or without thymic stromal lymphopoietin (TSLP), which is an effector of ILC2 survival.^22^ Throughout the 3 days of continuous observation, only a fraction of the hILC2 showed IL-5 and IL-13 secretion activity (Figure 4C). The time course for secretion activity from each activated hILC2 was unique; i.e., the start of secretion was widely distributed, ranging from hours to days after stimulation (Figure 4D). A statistical comparison of the secretion activity of the hILC2s between the donors showed contrasting effects for the TSLP; donor 1 showed a TSLP-dependent increase in the mean secretion amount, which was not observed in donor 2 (Figure 4E). Conversely, secretion-positive cells, which showed a significant difference between donors (Figure 4F; *p* < 0.001), were unaffected by TSLP stimulation in either donor (Figure 4F), indicating that the effect of TSLP on hILC2 from donor 1 was increasing the intensity of the secretion activity by individual cells but not increasing the number of cells secreting.

**Figure 5:**
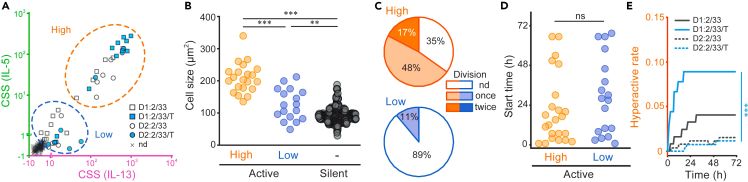
Phenotypic comparison among subpopulations of *ex vivo* hILC2s revealed by qLCI-S (A) Classification of activated ILC2 based on their levels of secretion signal. Comparison of the maximum CSS over time for IL-5 and IL-13 in each trace shown in Figure 3C; squares and circles indicate traces from active ILC2 of D1 and D2, respectively. Blue and white indicate the absence and presence of TSLP, respectively. Each CSS is plotted using axis with a separation occurring at CSS (IL-5) = 1 or CSS (IL-13) = 10. The data were plotted on the linear axis below the break and on the logarithmic axis above the break. A cross indicates traces below the threshold. The detectable trace data were divided into two groups using k-means clustering: hyperactive (high) and slightly active (low). (B) Comparison of the cell sizes among the three groups with different secretion activities. The average area of the cells obtained from the bright-field image observations of the last six time points in a 72-h period was used as a size parameter. Cells within each well were classified based on the CSS levels detected from each well, as shown in (C); when wells contained more than a single cell, their average area was adopted as a representative value. Differences were assessed using the Mann-Whitney U test (∗∗∗*p* < 0.001, ∗∗*p* < 0.01). (C) Comparison of the division activity between the two groups with different secretion activities. The number of divisions in the activated ILC2s was counted for 72 h using an AI-supported cell counter followed by visual verification. (D) Start of secretion comparisons for the two groups in active hILC2 with different secretion activities. There is no difference in the start of secretion between the hyperactive and slightly active hILC2s. Differences were assessed using the Mann-Whitney U test. (E) Comparison of the rate of activation of ILC2 resulting in hyperactivity. Differences were tested using a Benjamini-Hochberg corrected log rank multiple test (∗∗∗*p* < 0.001).

Next, we carefully compared the secretion activity tracks for the individual hILC2s and identified a pattern in the intensity differences and persistence of the secretion activity, similar to that observed in the mILC2 stimulated with IL-2 and IL-33. A classification depending on the CSS of individual hILC2s at 72 h after stimulation revealed the existence of three groups: hyperactive (high), slightly active (low), and silent (Figure 5A). In particular, the hyperactive hILC2s had other striking features, such as morphological enlargement (Figure 5B; *p* < 0.001) and high proliferative activity (Figure 5C). These differences in the secretory function may not be attributed to differences in response initiation, as the distribution when secretion first started did not differ between the slightly active and hyperactive hILC2s (Figure 5D). Focusing on the persistence of the secretion activity, the hyperactive hILC2s tended to have prolonged secretion activity, whereas the slightly active hILC2s tended to cease cytokine production within 10–30 h (Figure 4C). We statistically compared the secretion activity of the hyperactive hILC2s between donors. The results showed that the TSLP-stimulated donor 1-derived hILC2s had a significantly higher rate of hyperactive cells than those under other conditions, and the trend in the rates of the hyperactive cells was similar to that of the mean secretion amount (Figure 5E; *p* < 0.001). These results suggested that TSLP enhanced the hyperactivation of hILC2s, resulting in an increase in the total secretion amount as well as the number of hyperactive hILC2s at the population level, although the strength of the effect on hILC2 varied among donors.

In summary, qLCI-S revealed that the type 2 cytokine secretion following IL-33/TSLP stimulation in freshly isolated hILC2 was mediated only by a small but hyperactive subpopulation that had not previously been identified, which could also be referred to as an influential minority. Additionally, the expansion cultivation process emphasized this subpopulation, overshadowing the previously prevalent silent majority.

### Comparison of gene expression between hyperactive and silent ILC2s

**Figure 6:**
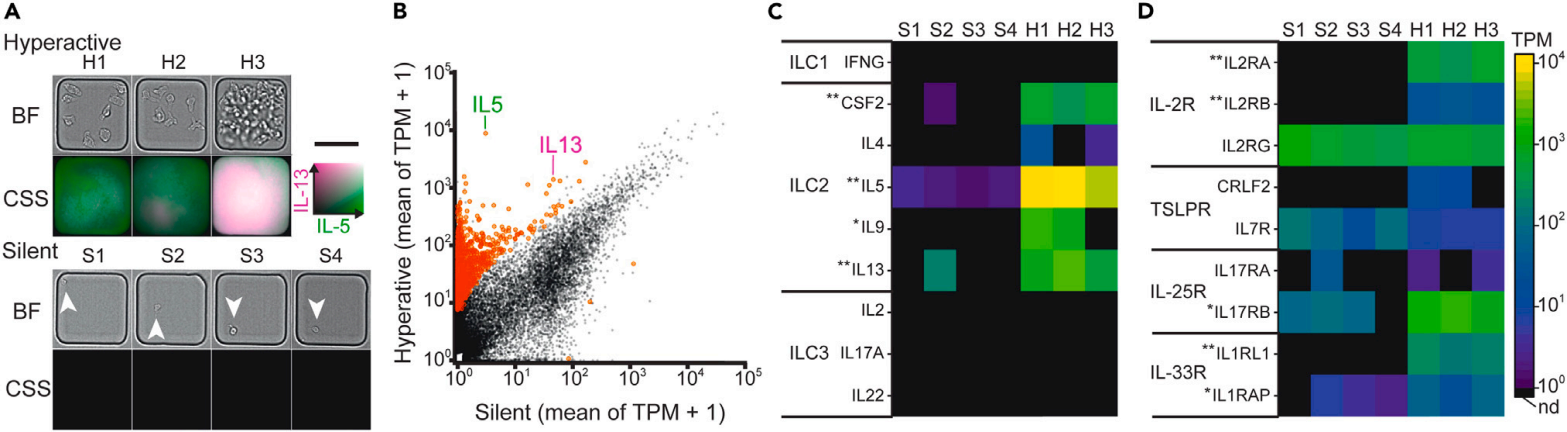
Functional transcriptome analysis of hILC2 (A) Images showing cell morphology (upper) and cumulative secretion activities (lower, IL-13: magenta, IL-5: green) of three hyperactive ILC2 clones (H1–H3) and four silent ILC2s with undetectable secretions (S1–S4) collected for RNA-Seq. Scale bar: 50 μm. (B) Scatterplot of mean transcripts per million (TPM) of the hyperactive (high) and silent ILC2s. DEGs with a *p* value <0.01 and false discovery rate (FDR) < 0.01 are colored in red. (C) Heatmap showing the expression levels of the characteristic cytokine genes of ILCs. (D) Heatmap showing the gene expression of the receptors required to activate the stimulation of ILC2s. Differences were tested using reproducibility-optimized test statistic (ROTS) analysis (∗∗∗*p* < 0.001, ∗∗*p* < 0.01 with FDR <0.05).

The hILC2 population was classified by qLCI-S into hyperactive, slightly active, and silent subpopulations according to the secretory function reflected in the phenotypic differences in secretion and proliferation activities and morphological changes. The comprehensive gene expression information underlying the differences in the secretory function promises to deepen our understanding of the regulatory mechanisms of hILC2 activation. We, therefore, tested the feasibility of cell selection and recovery based on the observed secretion activity and its application to single-cell transcriptome analysis. After 5 days of qLCI-S for the ILC2s stimulated with IL-2/IL-33/TSLPs, 3 clones of hyperactive and 4 single cells of silent phenotypes derived from a single donor were harvested (Figure 6A) and subjected to RNA sequencing (RNA-seq) analysis. Comparison of the mean transcripts per million (TPM) values for the hyperactive and silent phenotypes revealed the specific gene expression for each phenotype; e.g., the gene expression of IL-5 and IL-13 was significantly higher in the hyperactive group (Figure 6B and see the explanation of “DEG” in the STAR Methods). Conversely, we confirmed that the expression of a series of genes corresponding to the isolation markers of hILC2 used in this study was common in both groups (Figure S5A), ensuring that both could be regarded as hILC2s with completely different phenotypes.

To explore the origin of the different phenotypes reflected in the transcriptome, we investigated the genes differentially expressed between the hyperactive and silent ILC2s. For example, *CSF2*^23^ and *IL9*^23^^,^^24^ were predominantly expressed in hyperactive ILC2s as well as *IL-5* and *IL-13* (Figure 6C; *p* < 0.05 for *IL9* and *p* < 0.01 for others). *IL4* was slightly expressed in hyperactive ILC2s (Figure 6C), whereas ILC1 and ILC3 cytokine characteristics were not expressed in either the hyperactive or silent ILC2s. Furthermore, among the other gene characteristics of hILC2, the transcription factor *RORA*^24^ and the immune checkpoint protein *ICOS*^24^ tended to be highly expressed in hyperactive ILC2s (Figure S5B). These results indicated that silent ILC2s were not active, even at the transcriptional level. Based on this indication, the silent ILC2s were in a cellular state that was unresponsive to the applied stimuli. Therefore, we focused on gene expression of the receptors for hILC2-activating stimuli, compared the results between hyperactive and silent ILC2s, and found that the silent ILC2 barely expressed a subset of heteroreceptor genes for IL-2- and IL-33-activating stimuli (Figure 6D; *p* < 0.01). Among the three subunits of the IL-2R complex (alpha, beta, and gamma), IL-2Ra is known to be responsible for the affinity of IL-2 to its receptor. Additionally, ILC2s are known to upregulate IL-33 receptor expression encoded by *IL1RL1* upon stimulation with IL-33, resulting in increased IL-33 sensitivity. Therefore, it is conceivable that the differences in receptor expression at the transcription level may generate two distinct subpopulations, namely silent ILC2s and hyperactive ILC2s, as a positive feedback pathway. In future studies, applying this methodology across multiple donors will reveal common molecular mechanisms impacting cell secretion activity. The effectiveness of the functional transcriptome analysis based on qLCI-S presented here highlights the potential of qLCI-S to provide deeper insights in combination with various subsequent approaches for cellular characterization.

## Discussion

In this investigation, we established the qLCI-S platform, which integrates high-throughput, long-term, and dual-color quantitative tracking of secretion activity with transcriptomic analysis of identified phenotypic cells. Our ultimate goal with the development of qLCI-S is to further the discussion about whether cell kinetics after expanded culture accurately maintain their original nature reflecting *in vivo* conditions. This is crucial for understanding diverse immune responses among different donors at the cellular level. Applying the platform to primary cultures of hILC2s stimulated with IL-33 revealed that a minority of cells underwent repeated division and secreted substantial amounts of type 2 cytokines, ultimately forming the majority within the population. These activated cells, emerging from the minority, showed unique gene expression of stimulus receptors, differing from the silent cells. This finding highlights the tendency of expansion cultivation to focus predominantly on the active population, potentially overshadowing the silent majority. Supporting this, another study using LCI-S on hILC2s post-expansion culture showed many cells rapidly producing type 2 cytokines,^25^ indicating that hyperactive hILC2s proliferated and became the dominant group. These results affirm the importance of discerning differences between hyperactive and silent subsets for effective modulation of cellular activity and could greatly impact research on the secretory functions of rare cells.

The first challenge of this study involved verifying that temporal modifications in the time-resolved signal of the fluorescence immunospot assay on TIRFM were indicative of cellular secretion activity. Previously, LCI-S used a laser source for TIRFM,^16^ which impaired quantification by illuminating undesirable interference patterns due to coherence. To significantly improve the optical stability of the platform, we devised a fabrication process for an advanced microstructure of the nanoliter well array, using a layer of amorphous fluorocarbon polymer for refractive index coordination, which was sandwiched with a glass substrate and microfabricated polydimethylsiloxane. This structure allowed the use of TIRF illumination with incoherent LEDs^26^^,^^27^ in the LCI-S platform, ensuring the detection of sufficient fluorescence signal from the target at small incident light angles while reducing the background signal from light leakage. Consequently, due to the improved optical stability, the high spatiotemporal resolution of the qLCI-S platform enabled precise LCI-S quantitative analysis, revealing the previously elusive dynamic cytokine secretion in mILC2.

The second challenge of this study was establishing a configuration that would enable the quantitative tracking of the secretion activity over 5 days which could be achieved with a throughput that was sufficiently high to allow for statistical analysis. These measurement specifications for cytokine secretion were not previously reported with single-cell techniques.^15^ We guaranteed the ability of qLCI-S to measure cellular activity over several days without loss of activity in terms of resource availability needed for cellular function and phototoxicity by fluorescence imaging. For sensitive and quantitative detection of cytokine secretion, isolating the target cell in a closed nanoliter space (enclosed cultivation) is theoretically effective. However, the resources required by the cells are limited in the small volume of the nanoliter space. The effects of rapid environmental changes in the limited space associated with cell metabolism must also be considered.^28^ Previously, a volume-dependent decrease in antibody production was reported in enclosed cells.^29^ In contrast, isolating the target cell in an open-ended nanoliter space (semi-enclosed cultivation) maintained a constant environment, similar to traditional *in vitro* culture procedures. The validity of this method is also assured by the study from other perspectives in which simulations in open nanoliter space were performed using the immunospot method, which resulted in signal results that correlated with the secretion activity of the cells.^30^ It is important to note that even though semi-enclosed cultures are opened at the top, neighboring cells were restricted from communicating with each other because they were located at the bottom of deep wells. Measurement under this restriction was suitable for hILC2 in peripheral blood, where they have less opportunity to interact with each other due to their rarity *in vivo*. This advantage allowed us to reveal the stochastic nature of cytokine secretion initiation, as observed in both mouse ILC2s expanded in culture and freshly isolated human ILC2s. The timing of secretion initiation is widely distributed, ranging from 1 to 12 h (mostly around 2 h) for the mouse ILC2s, and from 1 to 72 h (mostly between 3 and 6 h) for the human ILC2s. On the other hand, hILC2s require cytokines such as IL-2, IL-33, and TSLP produced by other cells for their survival or activation, and the addition of these cytokines to the culture medium compensated for their needs. Thus, the strategy of using physical restriction of cell-cell communication and controllable pseudo-interactions by supplementation allows a clearer characterization of individual cells that retain their *in vivo* history. The stochastic nature of immune activation, in which the rate of activation in the population was reduced when the external stimulus concentration was low, has been reported in previous studies.^24^^,^^31^ However, in this study, we ensured that ILC2s were provided with a sufficient concentration of stimulus so that they were not depleted during long-term cultivation. Therefore, the stochastic nature of the activation exhibited by ILC2 was assumed to be controlled by an endogenous molecular mechanism that dispersed the timing of activation. In this study, we also identified a small subpopulation of hyperactive hILC2s with significant influence due to their dominant secretory and proliferative capabilities. Individual variations existed in the prevalence of these hyperactive hILC2s and their responsiveness to TSLP. These individual distinctions in cytokine secretion by hILC2s may contribute to differences in immune responses, including allergic predisposition. Investigating these individual differences in a larger and more diverse population is essential to elucidate the connection between individual cell secretion profiles and individual immune responses. Given the daily fluctuations in individuals' conditions, the parallelization of the qLCI-S platform is effective in acquiring multiple secretion response parameters in a single sample and monitoring them as individual conditions change. Even when focusing on rare peripheral blood cells, meaningful data can be derived from several hundred cells per condition, demonstrating that the approach is highly resource efficient. Increasing the degree of parallelization is a promising avenue for future development.

The third challenge of this study was implementing functional transcriptome analysis based on the phenotyping of secretory functions. The stimuli receptors, such as IL-2, IL-33, and TSLP, were highly expressed in hyperactive hILC2s and negatively regulated in silent hILC2. Since freshly purified ILC2s from human peripheral blood have low expression of the IL-33 receptor,^32^ functional fate determination of ILC2 activation and maintenance of hyperactivity presumably involve intracellular molecular mechanisms that control the promotion and maintenance of stimuli receptor expression. Comparing the dynamics of stimuli receptor expression and secretion activities would shed light on the detailed molecular mechanisms of ILC2 activation.

### Limitations of the study

qLCI-S also has some technical challenges that need to be addressed. The first is the limitations in the trade-off between throughput and temporal resolution. The throughput of qLCI-S depends on the size of the field of view of the objective lens for TIRFM and on the speed of scanning of the microscope system, which was approximately 4,000 shots/h in this study. This value implies that, for instance, consistent imaging every second is achievable solely when continuously monitoring secretion activity within a single field of view. However, when imaging two channels—bright field and secretion activity—the time lapse increases to approximately every 2 s. If the observation scope expands to 100 fields of view, the time interval extends to approximately every 200 s. Therefore, as the number of cells and parameters to be observed increases, the temporal resolution diminishes. Innovations such as widening the field of view, a faster microscope system control, and simultaneous multi-color imaging capabilities will improve the throughput and temporal resolution of qLCI-S. Second is the escape of cells from open-ended nanoliter wells. In this study, the high migration ability of mouse ILC2s made it challenging to evaluate long-term tracking over several days. Innovations in the structure of nanoliter wells are expected to interfere with the escape of cells from the wells. The third challenge is the multiplexing of measurement targets. Improvements are expected by increasing the variety of colors for different detection antibodies and the variety of capture antibodies via micropatterning of the solid-phase surface.

There exist certain inherent limitations that pose considerable challenges. As the methodology is grounded in the principles of immunoassay, the efficacy of this approach is contingent upon securing high-quality antibodies, given that detectability and sensitivity rely heavily on antibody affinity. In this regard, alternative immunoassay methods based on optical interference^33^ or plasmon-based assays,^34^ which directly detect antibody-antigen reactions without necessitating a detection antibody, offer broader application since they require only a single type of antibody, leading immediate detection without the need for deconvolution processing, unlike this study. Conversely, the sandwich immunoassay employed in our study using two distinct types of antibodies excels in specificity. Concerning sensitivity, our chosen detection principle, TIRFM, is well recognized for exceptional sensitivity, allowing detection down to a single molecule. Apart from this issue, a fundamental limitation is present. In scenarios marked by local cell accumulation, as observed in this study due to high proliferative capacity, or in situations where cells are closely adherent to each other, as is the case with epithelial cells, quantitative measurements become unfeasible. This is primarily attributed to the inaccessibility of secreted molecules and detection antibodies to the bottom surface surrounding the cell, which serves as the detection substrate.

Despite the limitations described earlier, qLCI-S has practical advantages, such as the simplicity of the measurement process, the use of commercially available antibodies, and the ease of integration with existing live-cell imaging techniques. qLCI-S may have applications like visualizing the secretion activities of various intercellular messengers such as cytokines, chemokines, and extracellular vesicles. In addition, DSS analysis, in principle, enables the interpretation of individual cell secretion activities in environments without nanoliter wells. This approach evokes the visualization of cell-cell communication mediated by secretion in densely populated cultures, such as those involving T cells and natural killer cells. Further innovations in qLCI-S and the development of applied research are expected to provide unprecedented perspectives in areas such as the elucidation of molecular mechanisms in diverse diseases, phenotypic drug discovery, provision of resources for regenerative medicine, and criteria for precision medicine.

## STAR★Methods

### Key resources table

**Table:** 

REAGENT or RESOURCE	SOURCE	IDENTIFIER
**Antibodies**		

Human/Mouse IL-5 Antibody	R&D systems	Cat#MAB405; RRID: AB_2233882
Mouse IL-5 Biotinylated Antibody	R&D systems	Cat#BAM705; RRID: AB_2127169
Mouse IL-13 Antibody	R&D systems	Cat#MAB413; RRID: AB_2124171
Mouse IL-13 Biotinylated Antibody	R&D systems	Cat#BAF413; RRID: AB_2124172
Human IL-5 Biotinylated Antibody	R&D systems	Cat#BAM6051; RRID: AB_356909
Human IL-13 Antibody	R&D systems	Cat#MAB213; RRID: AB_2124193
Human IL-13 Biotinylated Antibody	R&D systems	Cat#BAF213; RRID: AB_356264
FITC anti-human Lineage Cocktail (CD3, CD14, CD16, CD19, CD20, CD56)	Biolegend	Cat#348701; RRID: AB_10644012
Mouse PerCP/Cyanine5.5 anti-human CD45 Antibody	Biolegend	Cat#368504; RRID: AB_2566352
Mouse monoclonal PE anti-human CD127 (IL-7Ra) Antibody	Biolegend	Cat#351304; RRID: AB_10720185
Mouse monoclonal PE/Cyanine7 anti-human CD161 Antibody	Biolegend	Cat#339918; RRID: AB_11126745
Rat monoclonal Alexa Fluor 647 Anti-Human CD294 Antibody	Biolegend	Cat#558042; RRID: AB_2112699

**Chemicals, peptides, and recombinant proteins**		

Biolopidure-802	NOF	
dimethyl pimelimidate	Thermo Fisher Scientific	Cat#21666
CF660 streptavidin	Biotium	Cat#29040
Cy3 streptavidin	Thermo Fisher Scientific	Cat#438315
Fetal Bovine Serum	Japan Bioserum	Cat#S1820
recombinant mouse IL-2	R&D systems	Cat#402-ML
recombinant mouse IL-25	R&D systems	Cat#1399-IL
recombinant mouse IL-33	R&D systems	Cat#3626-IL
Imunace35 (recombinant human IL-2)	Shionogi	
recombinant human IL-25	R&D systems	Cat#1258-IL/CF
recombinant human IL-33	R&D systems	Cat#3625-IL/CF
recombinant human TSLP	R&D systems	Caat#1398-TS/CF
lymphoprep lhmphocyte separation medium	Axis-Shield	
Sylgard184	Dow Corning Toray	
CYTOP	AGC Chemicals	
SU-8 3050	Nippon Kayaku	
Vectabond reagent	Vector Laboratories	SP-1800
CT-solv 100E	AGC Chemicals	
CT-solv 180	AGC Chemicals	
dPEG4-biotin acid	Quanta Biodesign	Cat#10199
mineral oil	Sigma-Aldrich	Cat#M8410
Agencourt AMPure XP beads	Beckman Coulter	Cat#A63881

**Critical commercial assays**		

SMART-Seq v4 Ultra Low Input RNA Kit for Sequencing	Takara Bio	Cat#634891

**Deposited data**		

RNA-seq data	This study	GEO: GSE253100

**Experimental models: Cell lines**		

expansion-cultured fraction of ILC2 derived from mouse adipose tissue	Dr. Kazuyo Moro of RIKEN	Moro et al.^9^

**Software and algorithms**		

Nis-Elements AR	Nikon	version 4.6
Microsoft Excel	Microsoft	Office 2019
Bowtie2		version 2.3.1
RSEM software package		1.3.9
OriginPro	Origin Lab	2018

### Resource availability

#### Lead contact

Further information and requests for resources and reagents should be directed to and will be fulfilled by the lead contact, Yoshitaka Shirasaki (shirasaki@g.ecc.u-tokyo.ac.jp).

#### Materials availability

The TIRF-chip developed in this study is available from Live Cell Diagnosis, Ltd.

#### Data and code availability

• All RNA-seq data sets have been deposited at GEO and are publicly available as of the date of publication. Accession numbers are listed in the key resources table.
• This paper does not report original code.
• Any additional information required to reanalyze the data reported in this paper is available from the lead contact upon request.

### Experimental model and study participant details

#### Animal experiments

WT C57BL/6N mice were purchased from Charles River Laboratories Japan (Kanagawa, Japan). All the mice used in this research were retired female mice, and due to their status as retired breeders, their ages were unspecified. The animal experiments were conducted in accordance with the guidelines of the Institutional Animal Care and Use Committee of the RIKEN.

#### Human samples

Peripheral blood was obtained from three healthy volunteers: a 38-year-old male and a 32-year-old female for LCI-S observation, and a 33-year-old male for scRNA-seq following LCI-S observation, at Keio University School of Medicine. This study was approved by the Institutional Review Board of the Keio University School of Medicine (approval number: 20090009). All participants provided written informed consent.

### Method details

Antibodies, compounds, and cytokines.

Please see the key resources table.

#### Isolation of ILC2s from mouse mesentery

Mouse ILC2s were isolated from the mesenteric fat tissue of wild-type C57BL/6N female mice and enriched by autoMACS Pro Separator (Miltenyi Biotec, cat. no. 130-092-545) as Lin^-^ cells, and sorted by FACSAriaIII (BD Biosciences) as Lin^-^c-Kit^+^Sca-1^+^ cells as previously described.^35^

#### Isolation of ILC2s from human peripheral blood

hILC2s were obtained as described previously.^24^ Briefly, peripheral blood mononuclear cells (PBMCs) were separated using the Lymphoprep™ lymphocyte separation medium (Axis-Shield, Dundee, UK) according to the manufacturer’s protocols. PBMCs were stained with human lineage antibody cocktails (Lin) and anti-CD45, anti-CD127, anti-CRTH2, and anti-CD161 antibodies for 30 min at 2–8°C. ILC2s were sorted as PI-Lin-CD45^+^CD127+CRTH2+CD161+ cells via flow cytometry using MoFlo™ XDP (Beckman Coulter, Brea, CA, USA).

#### Optical arrangement

All measurements were performed with a completely automated inverted microscope (ECLIPSE Ti-E; Nikon, Tokyo, Japan) equipped with a high NA 60× objective lens (TIRF 60 × H; NA, 1.49; Nikon). The microscope was customized with the installation of white-light TIRF optics (high-performance Epi-fl illuminator module# TI-SFL) with LED light (X-Cite® XLED1, mounted with BGX:505-545 nm and RLX:615-655 nm; Excelitas Technologies Corp., Waltham, MA, USA). The laser light source used for comparison with the LED was purchased from Coherent (CUBE 640-40C; Santa Clara, CA, USA). Excitation filters (FF01-530/43 for Cy3 and FF01-635/18 for CF660R), emission filters (FF01-593/40 for Cy3 and FF01-692/40 for CF660R), and a dichroic mirror (FF560/659-Di01) were used. These optical filters were purchased from Semrock (Rochester, NY, USA). A semi-circular grinding acrylic lens block for measuring the TIR incident angle was purchased from KENIS Ltd. (H-100, refractive index 1.52; Osaka, Japan). Each image was projected onto a scientific CMOS camera (ORCA-Flash4.0 V2; Hamamatsu Photonics K.K., Shizuoka, Japan). A stage-top incubator (INUBG2TF-WSKM; Tokai Hit Co., Shizuoka, Japan) was used to control the temperature, humidity, and gas concentration. Control of the entire observation was performed using Nis-Elements AR 4.6 (Nikon).

#### Stable optical system design

We focused on the light source for TIRFM to establish a long-term stable observation platform because the coherent laser light (commonly used for TIRFM) generated an optical interference pattern variation that was the main cause of measurement noise in the multi-point time-lapse observations, as the stage movement caused a slight shift in the optical path. One limitation of this method is that the directivity of the LED light is lower than that of the laser. To establish the total internal reflection preventing the leakage of propagating light to the sample area, the minimum angle of the incident LED light must be greater than the critical angle at the interface of the glass and materials of the well microstructure. For a microstructure on a glass surface fabricated with PDMS, one of the most used materials for microfabricated chips employed in biological applications, the critical angle at the interface between the PDMS and glass is 67.1°, as calculated by sin^-1^(*n*_*PDMS*_/*n*_*Glass*_). Thus, the light incident at the critical angle on the interface between the aqueous solution and glass led to stray light generation in PDMS and high levels of background light (Figures S1A and S1B); therefore, the excitation light should be incident at a larger angle. In this case, the light from the LED with low directivity was blocked by the aperture limit of the objective lens (sin^-1^(NA_obj._/*n*_*Glass*_) = 78.6°), resulting in insufficient excitation intensity (Figure 1B). To overcome this issue using innovations in microfabrication structures, we developed a TIRF-dish in which a refractive index adjustment layer of an amorphous fluorocarbon polymer (AF layer; *n*_*AF*_ = 1.34) was inserted at the interface between the PDMS and glass. In the TIRF-dish, the critical angle for the total internal reflection was found to be sufficiently small (sin^-1^(*n*_*AF*_/*n*_*Glass*_) = 61.8°), thereby greatly improving the signal intensity and the signal-to-background ratio (Figure S1).

#### TIRF-dish fabrication

The TIRF-dish was composed of PDMS (Sylgard184; Dow Corning Toray, Tokyo, Japan), microscopic-grade coverslips (25 × 25 mm No. 1 cat# C025251, Matsunami Glass Ind., Osaka, Japan), and an amorphous fluorocarbon (AF) polymer (CYTOP; AGC Chemicals Company, Tokyo, Japan), which served as the reflection index matching layer with the aqueous solution. The TIRF-dish was prepared as follows: a mold pattern comprising 996 arrays that were 80 μm in diameter or had square through-holes with 115 μm center-to-center spacing and 80 μm thickness was fabricated on a Si wafer with SU-8 3050 (NIPPON KAYAKU, Tokyo, Japan) according to the manufacturer's instructions. PDMS (base: curing agent = 10:1) was poured onto the SU-8 mold and covered with a surface-inactivated glass slide. Then, the mold-PDMS-glass slide complex was pressed and cured at 80 °C for 1 h to form a through-hole array. The chamber block of the TIRF-dish was made with PDMS using a 3D fabricated mold (PEEK450G, Yasojima Proceed Co., Ltd., Osaka, Japan). The PDMS sheets and chamber blocks were immersed in n-hexane to remove any inhibitors of cellular activity. The cleaned PDMS sheet and chamber block were dried and permanently bonded using air plasma (SEDE-PFA; Meiwafosis Co., Ltd., Tokyo, Japan). The coverslip was cleaned by sonication in 5 N KOH for 1 h. The surface of the coverslip was aminated with VECTABOND reagent (SP-1800; Vector Laboratories, Burlingame, CA, USA), spin-coated with CYTOP 809A at 1,500 rpm for 20 s, and cured at 80°C for 1 h. Then, the PDMS sheet with the chamber block was permanently bound to the surface-modified CYTOP-coated coverslip and baked at 105°C for 3 h. The CYTOP coating exposed at the bottom of each nanoliter well was removed by wet etching using the CYTOP solvent (CT-solv100E and CT-solv180; AGC Chemicals Company) and cleaned by air plasma to expose the glass surface. Then, the bottom glass surface was aminated using the VECTABOND reagent. A mixture of capture antibodies (final concentration of 100 μg/mL) and dimethyl pimelimidate-2HCl (final concentration of 7 mg/ml) was loaded into each chamber to fix the capture antibodies on the bottom surface of each nanoliter well. The remaining reaction groups were blocked with monoethanolamine (0.1 M, pH 8.2). The TIRF-dish with immobilized antibodies was stored in phosphate-buffered saline supplemented with Lipidure BL802 reagent (0.2% w/v) at 4°C until further use.

#### Detection medium preparation

The biotin-labeled detection antibody was coupled with either CF660R-labeled streptavidin or Cy3-labeled streptavidin at 1:10 molar ratios in the dark for 3 h. Unoccupied sites on streptavidin were blocked with excess dPEG4-biotin acid (10199; Quanta BioDesign, Ltd., Powell, OH, USA). Unconjugated streptavidin and dPEG4-biotin were removed via ultrafiltration (Amicon Ultra-0.5, 100 kDa; Merck Millipore, Billerica, MA, USA). The detection media contained the prepared CF660R and Cy3-labeled detection antibodies for each antigen with final concentrations as follows: anti-mIL-5 (90 nM for Figure 1 and Video S1, 30 nM for others), anti-mIL-13 (30 nM), anti-hIL-5 (22.5 nM), anti-hIL-13 (90 nM), BSA (1% w/v), and the indicated combinations of the following additives (at final concentrations): rhIL-2 (20 U/ml), rhIL-25 (50 ng/ml), rhIL-33 (50 ng/ml), rhTSLP (50 ng/ml), rmIL-2 (10 ng/ml), rmIL-25 (10 ng/ml), and rmIL-33 (10 ng/ml).

Video S1. Visualization of dynamic IL-5 secretion activity of mILC2, related to Figure 1 Bright-field images of a mouse group 2 innate lymphoid cell (mILC2; upper left), cumulative secretion signal (CSS) images of IL-5 (lower left), and temporally deconvoluted secretion signal (DSS) images (lower right). The intensities of CSS and DSS are in pseudo-color, as shown on the color bar of each panel. The white outline in each panel indicates the cell shape in the bright-field image. The interval of each image is 1 min

#### Observation of type 2 response from individual ILC2s

An aliquot of 500 cultured mouse ILC2s or 100–300 freshly isolated human ILC2s was introduced into a chamber on a TIRF-dish and deposited in nanoliter wells via centrifugation (200 × g for 30 s). Accordingly, each nanoliter well contained zero to several mILC2s (bright-field in Figure 4B), and approximately 200–300 nanoliter wells containing single mILC2s were used for statistical analysis. In the hILC2 experiment, which was designed to detect rare cellular activity, all wells containing hILC2s were tracked during the 5-day monitoring period, and statistical analysis was performed using the total number of hILC2s at the initial time frame. The intensity values of the empty wells were used as references to compensate for the spatiotemporal changes in the excitation light intensity and to calculate the dynamic threshold. The culture supernatant was replaced with a freshly prepared detection medium immediately before observation. Mineral oil (M8410; Sigma-Aldrich) was layered on the detection medium to prevent evaporation. All wells in the TIRF-dish (996 wells × 4 chambers) were scanned using approximately 1-h cycles for 1 d or more. During the long-term tracking of hILC2s, data within 72 h were used for statistical analysis for the following reasons. First, there was a concern that the overproduction of cytokines following repeated division would be underestimated by the quantification due to the limited capacity of the capture antibody. Second, hILC2s that showed repeated divisions were observed to overflow from a well and migrate to neighboring wells.

### Quantification and statistical analysis

#### Calculation of the deconvoluted secretion signal (DSS) images

The spatiotemporally resolved DSS images were calculated using the image analysis software NIS-elements. First, pixel shifts of the images were aligned over time. Then the initial background was subtracted. The 5 frames rolling median was applied to reduce the noise before resizing by 5 × 5 pixel binning. The DSS image *DSS*_*t*_ was calculated by separating the influence of the probabilistic antibody binding kinetics from the cumulative secretion signal (CSS) image *CSS*_*t*_ as follows:

(Equation 1) $${DSS}_{t}=\left({CSS}_{t+1}-\sum_{i=0}^{t-1}{DSS}_{i}A_{t+1-i}\;\right)/A_{1}$$

where *i* = *nΔt*, and *A*_*t*_ was the normalized antibody kinetics given as follows:

(Equation 2) $$A_{t}=e^{-t/\tau_{1}}\left\{\alpha \left(1-e^{-t/\tau_{2}}\right)+\left(1-\alpha \right)\left(1-e^{-t/\tau_{3}}\right)\right\}$$

In the course of the calculation of Equation 1, whenever *DSS*_*t*_ yields a negative value, it is set to 0, reflecting the fact that secretion activity cannot be negative. This approach helps to mitigate noise that tends to amplify during the accumulation process. The time resolution of *Δt* = 5 frames was used in this experiment because the short time resolution did not provide a sufficient signal-to-noise ratio due to the small increase in signal.

In the Equation 2, $\tau_{1}$ was the decay constant which combination of stems from dissociation of antigen from capture antibody and photobleaching of detection antibody. The $\tau_{2}$ and $\tau_{3}$ were fast and slow rate constants of exponential association curve. The two binding rate constants reflected the polyclonal nature of the detection antibody.

We estimated each parameter for mouse IL-5 from standard curve mimicked by the instantaneous release of a recombinant protein by the microinjection method (Figure S6).

$$\tau_{1}=3.4\times 10^{4}\;\left[\min\text{.}\right],\tau_{2}=7.6\;\left[\min\text{.}\right],\tau_{3}=8.4\times 10^{1}\;\left[\min\text{.}\right],\alpha =0.599$$

For calculation of DSS image of the human IL-5, the normalized antibody kinetics *At* as follows:

(Equation 3) $$A_{t}=e^{-t/\tau_{1}}\left(1-e^{-t/\tau_{2}}\right)$$

where *τ*_*1*_ was the decay constant which probably stems from dissociation from capture antibody and photobleaching, *τ*_*2*_ was the association constant of the monoclonal detection antibody (Figure S7). We estimated each parameter using the microinjection method as follows:

$$\tau_{1}=2.8\times 10^{2}\;\left[h\right],\tau_{2}=0.27\;\left[h\right]$$

Then the DSS image of the human IL-5 was calculated according to Equation 1.

For calculation of DSS image of the human IL-13, we needed to consider cross-reaction of anti-human IL-13 antibody to the IL-5 immunocomplex, whereas the cross-reaction signal of anti-human IL-5 antibody to the IL-13 immunocomplex was empirically small enough to ignore. The cross-reaction signal of anti-human IL-13 antibody to the IL-5 immunocomplex ${Χ}_{t}^{13\to 5}$ at each t could be empirically compensated as follows:

(Equation 4) $${Χ}_{t}^{13\to 5}=\sum_{i=0}^{t}{DSS}_{i}^{IL-5}\chi \left({\Delta t}_{i}\right)$$

(Equation 5) $${\chi \left(\Delta t\right)=\beta e}^{-\Delta t/\tau_{4}}\left\{\alpha \left(1-e^{-\Delta t/\tau_{5}}\right)+\left(1-\alpha \right)\left(1-e^{-\Delta t/\tau_{6}}\right)\right\}$$

We estimated each parameter using the microinjection method as follows:

$$\tau_{4}=2.8\times 10^{2}\;\left[h\right],\tau_{5}=0.23\;\left[h\right],\tau_{6}=2.5\times 10\;\left[h\right]$$

α=0.23,β=0.33

Then we calculated CSS of IL-13 signal as follows:

(Equation 6) $${CSS}_{t}^{IL-13}={FI}_{t}^{IL-13}-{Χ}_{t}$$

For calculation of DSS image of the human IL-5, the normalized antibody kinetics *At* as follows:

(Equation 7) $$A_{t}=\left\{\alpha e^{-\;t/\tau_{1}}+\left(1-\alpha \right)e^{-\;t/\tau_{2}}\right\}\left(1-e^{-\;t/\tau_{3}}\right)$$

We estimated each parameter using the microinjection method as follows:

$$\tau_{1}=0.84\;\left[h\right],\tau_{2}=2.1\times 10^{2}\;\left[h\right],\tau_{3}=1.0\;\left[h\right]$$

α=0.71

Then the DSS image of the human IL-13 was calculated according to Equation 1.

#### Image analysis for statistical comparison of high-throughput qLCI-S measurement

The secretion activity of the cells in each well was analyzed using an in-house VBA program in Microsoft Excel using the mean intensities and cell numbers of each well at each time frame. The mean intensity of each well was measured from IL-5 and IL-13 secretion signal images using commercially available image analysis software (NIS-elements AR 4.6, Nikon). The number of cells in each well was counted manually or estimated by an in-house artificial intelligence algorithm.^36^ First, we corrected the intensity deviation between the wells for the field-of-view configuration and the time period. The mean fluorescence intensities of empty wells were summarized and averaged for each configuration (upper left, upper right, lower left, and lower right in the field of view), each chamber, and each time frame. The quotients of these values to the total average were calculated as coefficients, and the intensities of the individual wells were corrected using these coefficients. Next, we subtracted the initial intensity from the intensity at each time frame for each well. For wells with an initial intensity outlier larger than 1.5 × (interquartile range), the average intensity of empty wells was subtracted. This calculation eliminates the contribution of autofluorescence from infrequent contaminants and well walls in each well. Third, the influence of antibody staining kinetics and cross-staining of IL-5 and IL-13 was removed by temporal deconvolution. The calculated values, which were filtered by temporal integration and moving the median to reduce the processing noise, were then used for statistical evaluation of the secretion activity of individual cells.

The detection of secretion positivity for the intensity of each well was performed using a detection limit [3 × standard deviation (SD)] for the intensity of the empty wells. This SD, however, was estimated using the median absolute deviation of the empty wells multiplied by 1.4826. Additionally, owing to the continuity of the accumulating signal in the temporal direction, we judged a secretion signal trajectory to be positive if it was above the threshold of ≥ 2 time frames, while allowing for a transient fall below the threshold if it was within three time frames. The determination of hyperactive hILC2 was performed in conjunction with the above secretion positivity using a threshold of 50× SD corresponding to the results of the k-means cluster analysis (Figure 5A).

#### Functional RNA-Seq on LCI-S

ILC2s were retrieved from the TIRF-dish using a glass capillary (L-Tip 15 mm 60° 15 mm, Yodaka Co., Ltd., Kanagawa, Japan) controlled by a pneumatic microinjector (IM-11-2, Narishige, Tokyo, Japan) and a micromanipulator (Transferman NK2, Eppendorf, Hamburg, Germany) and transferred into 4 ml RNase-free water (06442-95, Nacalai Tesque, Kyoto, Japan) in 0.2-ml polymerase chain reaction tubes. Isolated cells were spun down, flash-frozen in liquid nitrogen, and stored at −80 °C until further use. We obtained cDNA from poly(A) RNA of each sample using a SMART-Seq v4 Ultra Low Input RNA Kit for Sequencing (Takara Bio Inc., Shiga, Japan), following the manufacturer’s instructions. The pre-amplified cDNA was obtained after reverse transcription (90 min at 42°C, 10 min at 70°C) and pre-amplification (24 cycles (N1–N4) or 18 cycles (P1–P3): 1 min at 95°C, 10 s at 98°C, 30 s at 65°C, and 3 min at 68°C). After the purification of the amplified cDNA using Agencourt AMPure XP beads (A63881, Beckman Coulter), cDNA sequences were analyzed using Illumina HiSeq with an entrusted analysis service (50 bp single-read, Medical & Biological Laboratories Co., Ltd. Japan). The obtained sequence data were mapped to the reference genome (GRCh38.87) using Bowtie2 (version 2.3.1), and the number of transcripts was counted using the RSEM software package (1.3.0). Subsequent ROTS analysis of the expressed genes with TPM ≥ 1 from one or more samples detected 1,869 differentially expressed genes (DEGs). The distribution of the TPMs calculated from the count data for each cell showed a continuous distribution in hyperactive ILC2, whereas the distribution obtained from silent ILC2 showed a sharp decrease in the number of genes with TPMs below 50 (Figure S8). This phenomenon is seen when cDNA amplification is performed using a low absolute number of transcripts and is caused by limits in the reverse transcription and amplification efficiency. We found no significant influence of the cells recovered from the qLCI-S platform, allowing us to obtain data comparable to normal single-cell RNA-Seq. The detection threshold was set at 44.7 (Figure S8), and significant difference analysis was performed for genes with TPM values above the threshold in the hyperactive ILC2s.

#### Statistical analysis

The secretion signals were statistically analyzed using the Mann–Whitney or log-rank tests. The false discovery rate of each test was controlled using the Benjamini–Hochberg procedure. Statistical significance was set at p < 0.05. These graphs were drawn, and the tests were performed using the statistical software OriginPro (Origin Lab, Northampton, MA, USA).

## References

[1] Armingol E., Officer A., Harismendy O., Lewis N.E. (2021). Deciphering cell–cell interactions and communication from gene expression. Nat. Rev. Genet..

[2] Clark H.F., Gurney A.L., Abaya E., Baker K., Baldwin D., Brush J., Chen J., Chow B., Chui C., Crowley C. (2003). The secreted protein discovery initiative (SPDI), a large-scale effort to identify novel human secreted and transmembrane proteins: A bioinformatics assessment. Genome Res..

[3] van Niel G., D’Angelo G., Raposo G. (2018). Shedding light on the cell biology of extracellular vesicles. Nat. Rev. Mol. Cell Biol..

[4] van Gorp H., van Opdenbosch N., Lamkanfi M. (2019). Inflammasome-dependent cytokines at the crossroads of health and autoinflammatory disease. Cold Spring Harb. Perspect. Biol..

[5] Galdiero M.R., Marone G., Mantovani A. (2018). Cancer inflammation and cytokines. Cold Spring Harb. Perspect. Biol..

[6] Nedeva C., Menassa J., Puthalakath H. (2019). Sepsis: Inflammation is a necessary evil. Front. Cell Dev. Biol..

[7] Fajgenbaum D.C., June C.H. (2020). Cytokine storm. N. Engl. J. Med..

[8] Fafián-Labora J.A., O’Loghlen A. (2020). Classical and nonclassical intercellular communication in senescence and ageing. Trends Cell Biol..

[9] Moro K., Yamada T., Tanabe M., Takeuchi T., Ikawa T., Kawamoto H., Furusawa J.I., Ohtani M., Fujii H., Koyasu S. (2010). Innate production of TH2 cytokines by adipose tissue-associated c-Kit+Sca-1+ lymphoid cells. Nature.

[10] Kabata H., Moro K., Fukunaga K., Suzuki Y., Miyata J., Masaki K., Betsuyaku T., Koyasu S., Asano K. (2013). Thymic stromal lymphopoietin induces corticosteroid resistance in natural helper cells during airway inflammation. Nat. Commun..

[11] Drake L.Y., Kita H. (2017). IL-33: biological properties, functions, and roles in airway disease. Immunol. Rev..

[12] von Moltke J., Ji M., Liang H.E., Locksley R.M. (2016). Tuft-cell-derived IL-25 regulates an intestinal ILC2-epithelial response circuit. Nature.

[13] Cayrol C., Girard J.-P. (2018). Interleukin-33 (IL-33): A nuclear cytokine from the IL-1 family. Immunol. Rev..

[14] Mjösberg J.M., Trifari S., Crellin N.K., Peters C.P., van Drunen C.M., Piet B., Fokkens W.J., Cupedo T., Spits H. (2011). Human IL-25-and IL-33-responsive type 2 innate lymphoid cells are defined by expression of CRTH2 and CD161. Nat. Immunol..

[15] Yamagishi M., Ohara O., Shirasaki Y. (2020). Microfluidic immunoassays for time-resolved measurement of protein secretion from single cells. Annu. Rev. Anal. Chem..

[16] Shirasaki Y., Yamagishi M., Suzuki N., Izawa K., Nakahara A., Mizuno J., Shoji S., Heike T., Harada Y., Nishikomori R., Ohara O. (2014). Real-time single-cell imaging of protein secretion. Sci. Rep..

[17] Murai S., Yamaguchi Y., Shirasaki Y., Yamagishi M., Shindo R., Hildebrand J.M., Miura R., Nakabayashi O., Totsuka M., Tomida T. (2018). A FRET biosensor for necroptosis uncovers two different modes of the release of DAMPs. Nat. Commun..

[18] Polykratis A., Martens A., Eren R.O., Shirasaki Y., Yamagishi M., Yamaguchi Y., Uemura S., Miura M., Holzmann B., Kollias G. (2019). A20 prevents inflammasome-dependent arthritis by inhibiting macrophage necroptosis through its ZnF7 ubiquitin-binding domain. Nat. Cell Biol..

[19] Liu T., Yamaguchi Y., Shirasaki Y., Shikada K., Yamagishi M., Hoshino K., Kaisho T., Takemoto K., Suzuki T., Kuranaga E. (2014). Single-cell imaging of caspase-1 dynamics reveals an all-or-none inflammasome signaling response. Cell Rep..

[20] Loeffler D., Schroeder T. (2019). Understanding cell fate control by continuous single-cell quantification. Blood.

[21] Skylaki S., Hilsenbeck O., Schroeder T. (2016). Challenges in long-term imaging and quantification of single-cell dynamics. Nat. Biotechnol..

[22] Camelo A., Rosignoli G., Ohne Y., Stewart R.A., Overed-Sayer C., Sleeman M.A., May R.D. (2017). IL-33, IL-25, and TSLP induce a distinct phenotypic and activation profile in human type 2 innate lymphoid cells. Blood Adv..

[23] Mjösberg J., Bernink J., Golebski K., Karrich J.J., Peters C.P., Blom B., te Velde A.A., Fokkens W.J., van Drunen C.M., Spits H. (2012). The transcription factor GATA3 is essential for the function of human type 2 innate lymphoid cells. Immunity.

[24] Wagner M., Moro K., Koyasu S. (2017). Plastic heterogeneity of innate lymphoid cells in cancer. Trends Cancer.

[25] Tanaka Y., Yamagishi M., Motomura Y., Kamatani T., Oguchi Y., Suzuki N., Kiniwa T., Kabata H., Irie M., Tsunoda T. (2023). Time-dependent cell-state selection identifies transiently expressed genes regulating ILC2 activation. Commun. Biol..

[26] Kogel A., Kalwa H., Urban N., Schaefer M. (2019). Artifact-free objective-type multicolor total internal reflection fluorescence microscopy with light-emitting diode light sources—Part I. J. Biophotonics.

[27] Axelrod D. (2001). Total internal reflection fluorescence microscopy in cell biology. Traffic.

[28] Molter T.W., McQuaide S.C., Holl M.R., Meldrum D.R., Dragavon J.M., Anderson J.B., Young A.C., Burgess L.W., Lidstrom M.E. (2008). A new approach for measuring single-cell oxygen consumption rates. IEEE Trans. Autom. Sci. Eng..

[29] Isozaki A., Nakagawa Y., Loo M.H., Shibata Y., Tanaka N., Setyaningrum D.L., Park J.W., Shirasaki Y., Mikami H., Huang D. (2020). Sequentially addressable dielectrophoretic array for high-throughput sorting of large-volume biological compartments. Sci. Adv..

[30] Torres A.J., Hill A.S., Love J.C. (2014). Nanowell-based immunoassays for measuring single-cell secretion: Characterization of transport and surface binding. Anal. Chem..

[31] Wada T., Hironaka K.i., Kuroda S. (2021). Cell-to-cell variability serves as information not noise. Curr. Opin. Syst. Biol..

[32] Boberg E., Johansson K., Malmhäll C., Calvén J., Weidner J., Rådinger M. (2020). Interplay Between the IL-33/ST2 Axis and Bone Marrow ILC2s in Protease Allergen-Induced IL-5-Dependent Eosinophilia. Front. Immunol..

[33] McDonald M.P., Gemeinhardt A., König K., Piliarik M., Schaffer S., Völkl S., Aigner M., Mackensen A., Sandoghdar V. (2018). Visualizing Single-Cell Secretion Dynamics with Single-Protein Sensitivity. Nano Lett..

[34] Li X., Soler M., Szydzik C., Khoshmanesh K., Schmidt J., Coukos G., Mitchell A., Altug H. (2018). Label-Free Optofluidic Nanobiosensor Enables Real-Time Analysis of Single-Cell Cytokine Secretion. Small.

[35] Moro K., Ealey K.N., Kabata H., Koyasu S. (2015). Isolation and analysis of group 2 innate lymphoid cells in mice. Nat. Protoc..

[36] Kamatani T., Fukunaga K., Miyata K., Shirasaki Y., Tanaka J., Baba R., Matsusaka M., Kamatani N., Moro K., Betsuyaku T., Uemura S. (2017). Construction of a system using a deep learning algorithm to count cell numbers in nanoliter wells for viable single-cell experiments. Sci. Rep..

