# Supplementary material for: Quantitative live-cell imaging of secretion activity reveals dynamic immune responses
Source: iScience. 2024 Apr 27;27(6):109840. doi: 10.1016/j.isci.2024.109840 (PMC11109006; doi:10.1016/j.isci.2024.109840)
Supplement: Document S1. Figures S1–S8 [file mmc1.pdf]

iScience, Volume *27*

Supplemental information

Quantitative live-cell imaging of secretion activity reveals dynamic immune responses

Mai Yamagishi, Kaede Miyata, Takashi Kamatani, Hiroki Kabata, Rie Baba, Yumiko Tanaka, Nobutake Suzuki, Masako Matsusaka, Yasutaka Motomura, Tsuyoshi Kiniwa, Satoshi Koga, Keisuke Goda, Osamu Ohara, Takashi Funatsu, Koichi Fukunaga, Kazuyo Moro, Sotaro Uemura, and Yoshitaka Shirasaki

Supplemental information

Figures S1 to S8

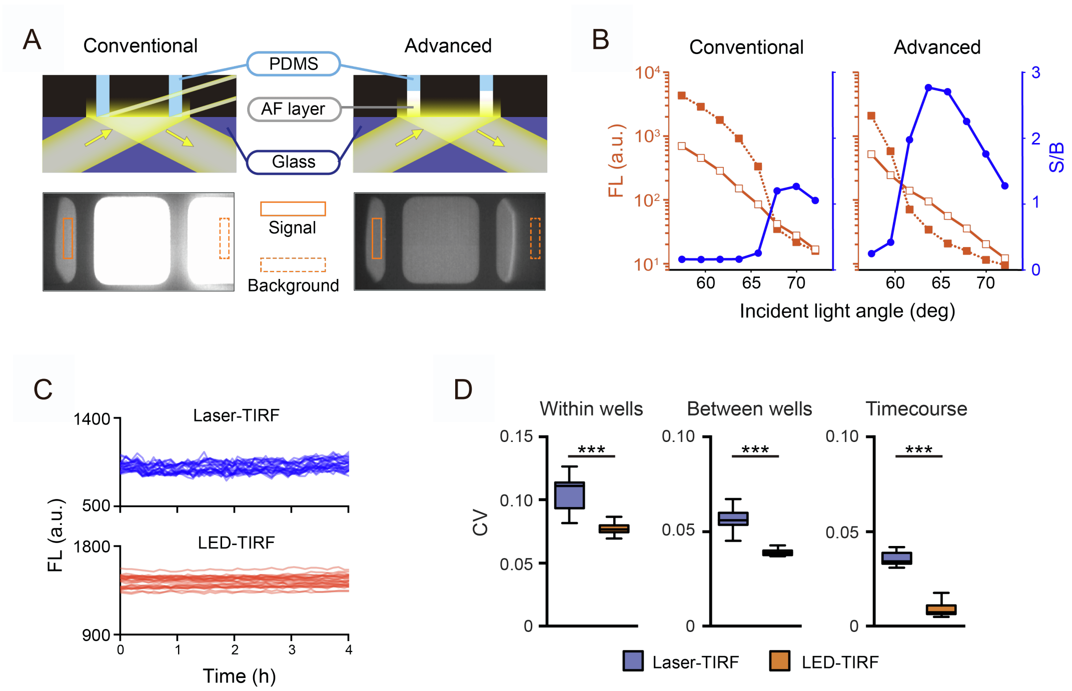

Figure S1: Stabilization of TIRF illumination for spatiotemporal noise reduction, Related to Figure 1

(A) Illustration of the optical stability of the qLCI-S platform. The advanced microfabricated well structure consisted of a bottom glass substrate and a nanoliter well array composed of polydimethylpolysiloxane (PDMS) with a refractive index-adjusting layer of amorphous fluorocarbon polymer (AF layer) sandwiched in between. The fluorescent signal of the detection antibody floating in the evanescent light field is indicated by the "Signal" area (solid line). The bright fluorescent signal of the detected antibody in stray light leaking from the interface between the glass and PDMS in the conventional structure is shown in the "Background" area (dashed line). The AF layer inserted into the interface between the glass and PDMS prevents the generation of stray light. (B) Effects of the AF layer on improving the ratio of signal-to-background noise. Changes in the signal intensity (red open squares, mean intensity of the area with no stray light overlapping, outlined by solid lines and indicated as "Signal" in (A)) and background noise (red filled squares; mean intensity of the area illuminated only with stray light, outlined by dash lines and indicated as "Background" in (A)) along with the angles of the incident excitation light without (left) or with (right) the AF layer were plotted. The signal-to-noise ratio (blue-filled circles) was calculated from these values. (C) Comparison of the temporal stability of fluorescent signals with the Laser-TIRF and

LED-TIRF illuminators. The traces of mean fluorescent intensity of individual wells illuminated with the Laser-TIRF (upper panel, blue lines) and LED-TIRF (lower panel, red lines) illuminators are shown. (D) Comparison of the spatiotemporal variation of the TIRF signals. The left panel shows the coefficient of variation (CV) of intensities within the wells at each time point, which reflected the intensity variation of the interference pattern of the laser or LED light. The middle panel shows the CV between wells at each time point. The right panel shows the CV of the temporal fluctuations of each well. Differences were assessed using the Mann–Whitney *U* test (*** $p < 0.001$).

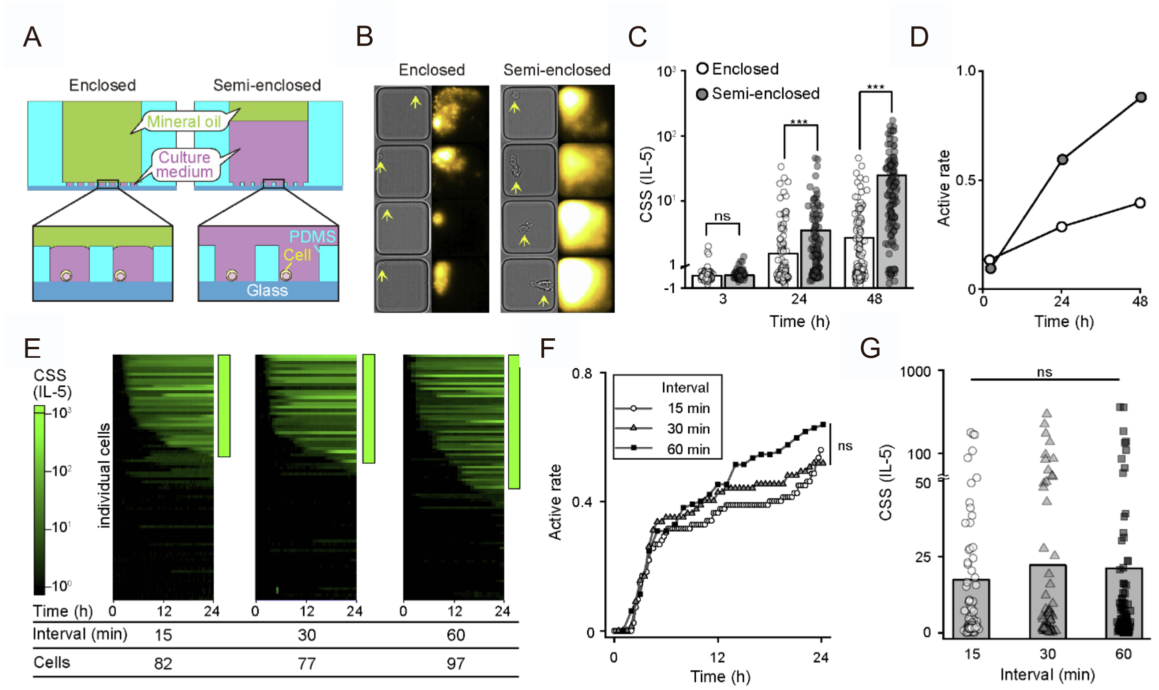

Figure S2: Evaluation of the qLCI-S platform, Related to Figure 1

(A) Schema of the experimental setup for the single-cell cultivation with the nanoliter well array. For enclosed cultivation, cells (yellow) were sealed in nanoliter wells with mineral oil (green) along with a limited volume of culture medium (purple); for semi-enclosed cultivation, cells were deposited in open-ended nanoliter wells surrounded by a large pool of culture medium. (B-D) Comparison of IL-5 secretion activity between the enclosed and semi-enclosed cultivation. (B) The representative images of the secretion activity under the two types of cultivation 48 h after isolation. Yellow arrows indicate the position of the cells. Comparison of the cumulative secretion signal (CSS) from individual cells (C) and the activation rate of mILC2s (D) in each cultivation state. In (C), each CSS is plotted using axis with a separation occurring at CSS (IL-5) = 1. The data were plotted on the linear axis below the break and on the logarithmic axis above the break. *** $p < 0.001$ or ns $p > 0.05$; non-parametric Mann–Whitney test between the two cultivation states. (E) Chronological heatmap of the CSS of individual cells tracked by qLCI-S for the indicated number of cells with the indicated time-lapse intervals. The color reflects the CSS intensity. Each row reflects the dynamic secretion activity of an individual cell. The color scale bar shows the intensity of IL-5 secretion signals. Secretory positive cells are indicated by green lines on the right side of the chronological heatmap. (F) Rate of activation of mILC2s at different time-lapse intervals. Differences were tested using the log-rank test. (G) Maximum CSS of each cell with different time-lapse intervals. Differences were assessed using the Mann–Whitney *U* test.

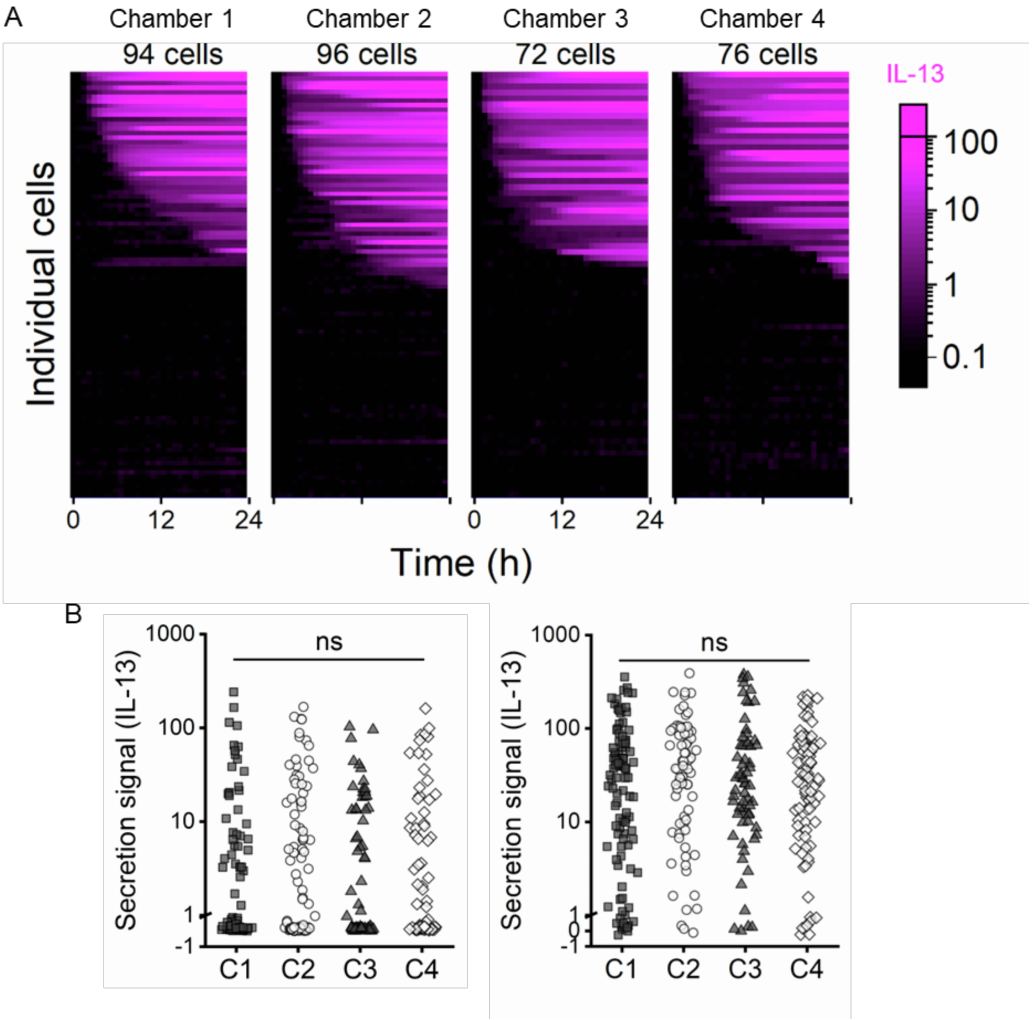

Figure S3: Evaluation of the reproducibility and independence of the four chambers of the TIRF-dish, Related to Figure 2

(A) Chronological table of the cumulative secretion signals of every single cell arranged for each sample chamber (C1–C4) with the same culture environment. Each row reflects the dynamic activity of an individual cell. Color scale bars show the intensity of IL-13 secretion signals. (B) Maximum cumulative secretion signal (CSS) of mILC2 introduced into each of the four sample chambers (C1-C4) of the TIRF-chip under uniform IL-33 conditions (left) and IL-2/IL-33 stimulation (right). The signal was depicted using the CSS (IL-13) = 1 separation axis, employing a linear scale below the separation point and a logarithmic scale above it. Statistical differences were assessed using the Mann-Whitney *U* test, indicating significance at $p<0.05$. 'ns' indicates non-significance.

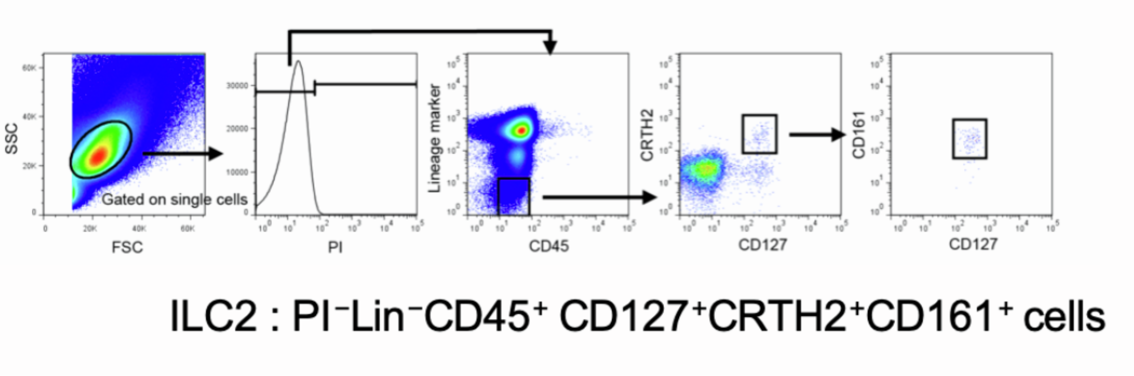

Figure S4: Purification of human peripheral ILC2s, Related to Figure 4

Fluorescence flow cytometry plots showing gating strategy for sorting human ILC2s. Peripheral blood mononuclear cells were isolated using Lymphoprep and labeled with several surface differentiation markers, as described in the Materials and Methods. The labeled cells were sorted via fluorescence-assisted cell sorting (FACS) and identified as ILC2s.

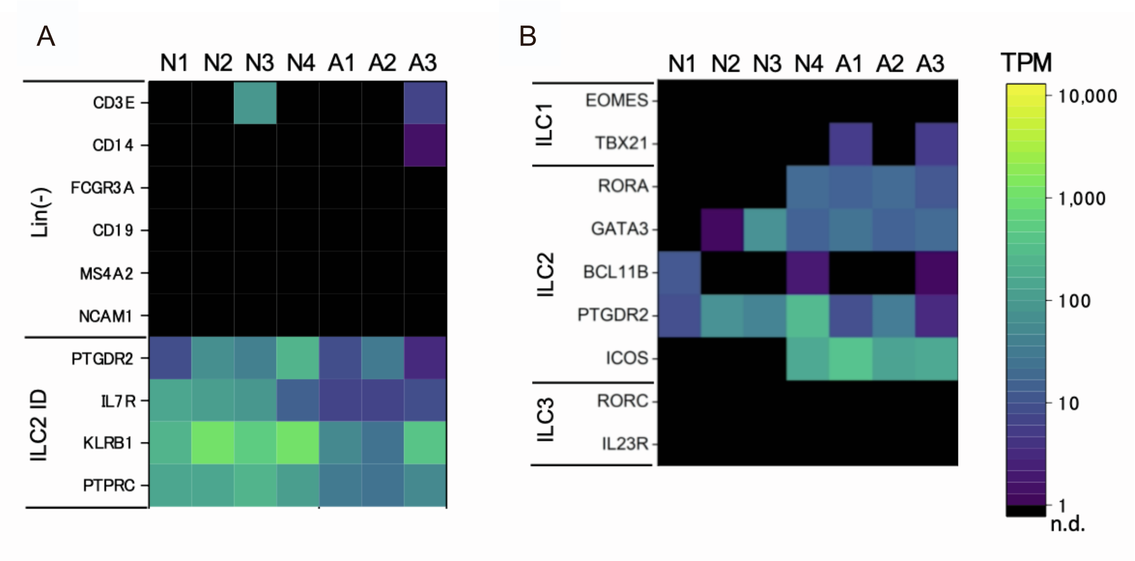

Figure S5: Expression differences of active and silent ILC2s, Related to Figure 6

(A) Transcript expression of genes used for FACS purification showed in parallel to proteins. Lineage markers were rarely detected from both active and silent populations. In contrast, identity markers for ILC2 were detected not only from active populations but also specifically detected from silent populations exceeding the confidence level. (B) Comparisons of the expression of specific genes of each ILC type. ILC2-specific genes were detected in both silent and active populations, except for BCL11B. Expression of genes specific to ILC1 and ILC3 was rarely detected.

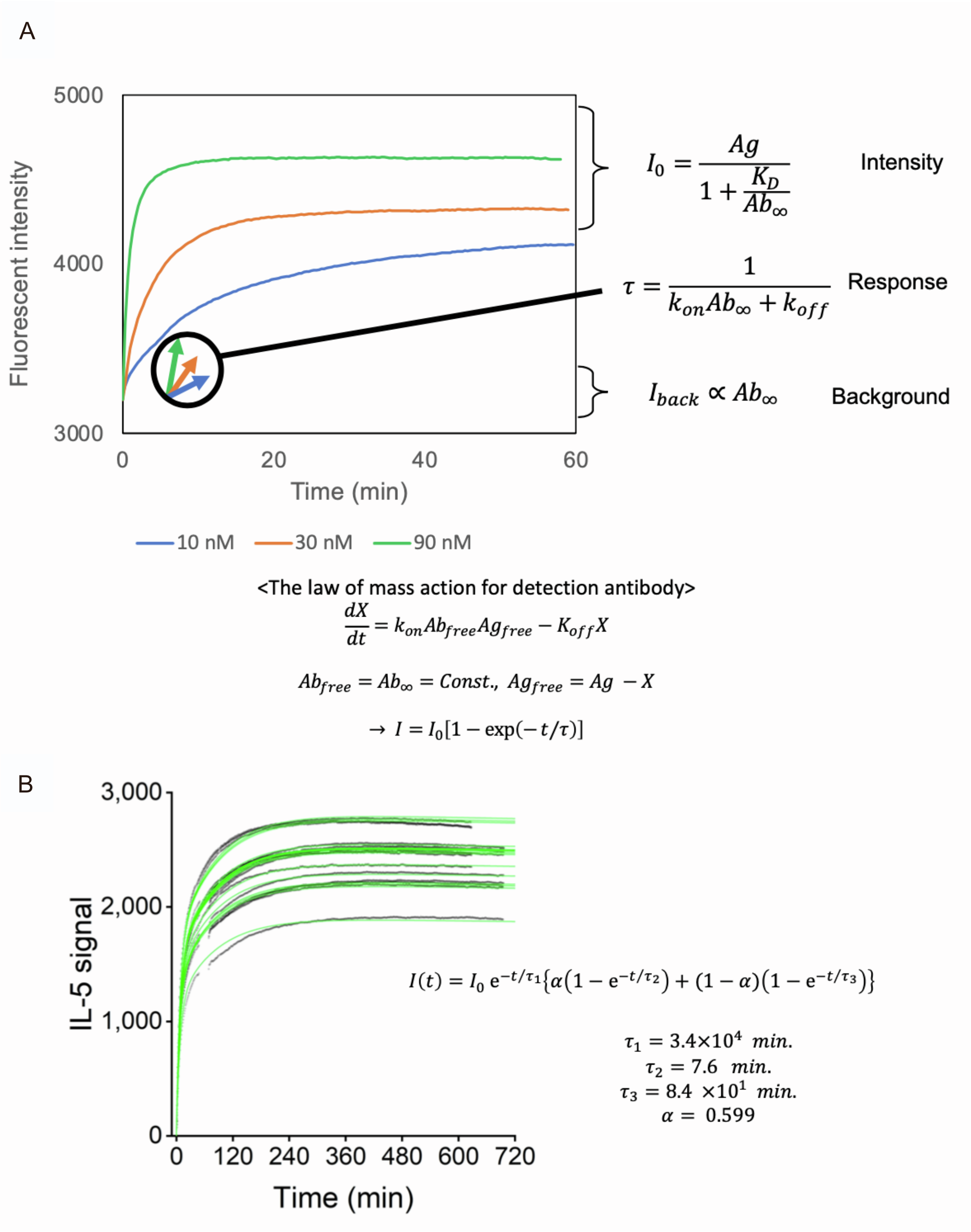

Figure S6: Binding dynamics of detection antibody, Related to STAR Methods

The typical binding dynamics of the fluorescent sandwich immunocomplex are shown. In this assay, transient secretion activity was mimicked by the instantaneous release of a recombinant protein by microinjection. A high local concentration of the capture antibody enables the system to immediately immobilize the secreted antigen compared to that in

the measurement interval of the time-lapse. Immediate immobilization is also reflected in the localized pattern of the secretion signal. Compared with the fast dynamics of capturing, the staining dynamics using the detection antibody require time to reach equilibrium. The law of mass action for single-step reactions explains the dynamics of the time-resolved fluorescence immunoassay used in LCI-S. (A) Demonstration of a time-resolved fluorescence immunoassay for human IL-5. IL-5 (100 ng/mL) was captured on an anti-human IL-5 antibody-coated glass bottom. After washing out excess unbound IL-5, TIRF measurements were performed. The intensities increased immediately after the addition of detection antibodies at a final concentration of 10, 30, or 90 nM. The maximal intensity was controlled by the efficiency of forming an immunocomplex at equilibrium, depending on the amount of antigen and the affinity and concentration of the detection antibody. The response speed was controlled by the affinity and concentration of the detection antibodies. The background intensity is proportional to the concentration of the detection antibody, and the detection sensitivity generally decreases in proportion to the square root of the background light intensity. The cost of measurement increased in proportion to the concentration of the detection antibody. (B) Time-resolved fluorescence immunoassay every 1 min for recombinant mouse IL-5 in different amounts microinjected into a nanoliter well in a TIRF-dish with 90 nM of anti-mouse IL-5 detection antibody. The curves of the formula indicated on the graph fit well with the data. The first term with τ_1 indicates the exponential decay of the secretion signal; the second term with τ_2 and the third term with τ_3 indicate the fast and slow exponential growth of the secretion signal with ratio α, respectively. The fitted parameter was used to calculate the secretion activity (Fig. 2).

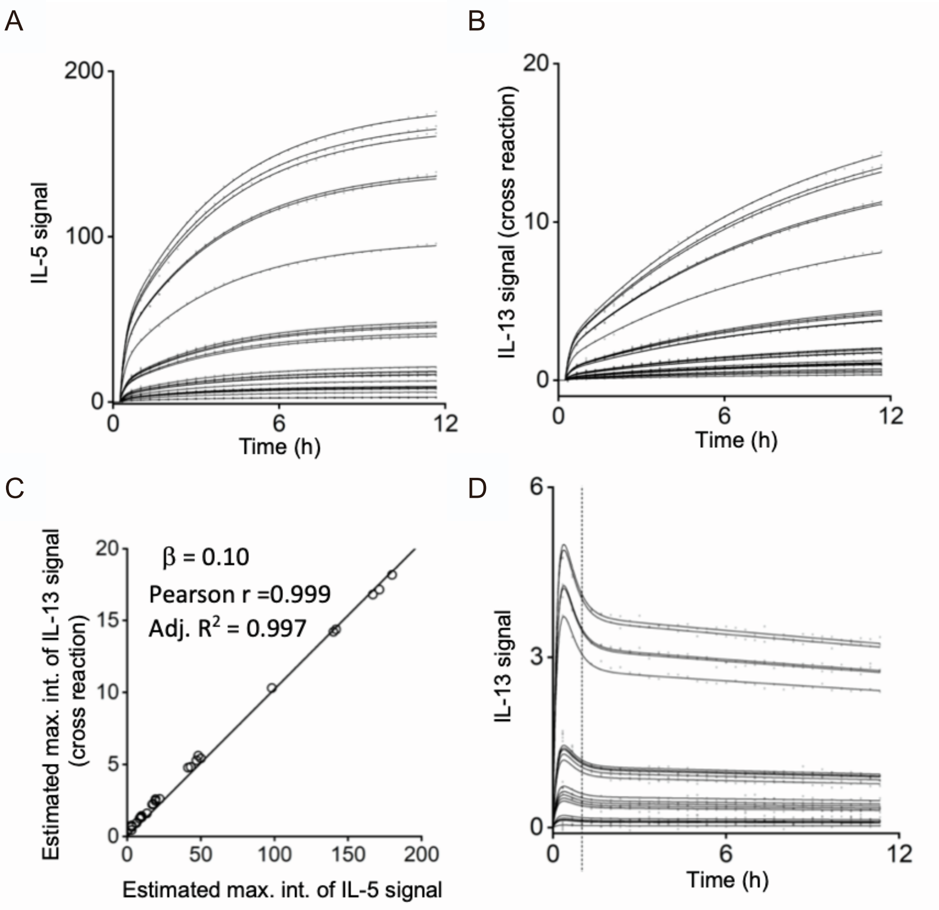

$$I_{IL-5} = I_0 e^{-t/\tau_c}\{\alpha(1 - e^{-t/\tau_{fast}}) + (1-\alpha)(1 - e^{-t/\tau_{slow}})\}$$

I_0: IL − 5 *maximum intensity*, α: ratio between faster and slower decay = 0.71
τ_c: time constant of decay = 6.8 × 10^{25} [h]
τ_{fast}: time constant of faster staining=0.18 [h], τ_{slow}: time constant of slower staining=3.8 [h]

$$I_{cross\ to\ IL-13} = I_0 \beta e^{-t/\tau_c}\{\alpha(1 - e^{-t/\tau_{fast}}) + (1-\alpha)(1 - e^{-t/\tau_{slow}})\}$$

I_0: IL − 5 *maximum intensity*, τ_c: time constant of decay = 2.2 × 10^{20} [h]
τ_{fast}: time constant of faster staining=0.17 [h], τ_{slow}: time constant of slower staining=8.2 [h]
β: cross reaction factor =0.1, α: ratio between faster and slower on staining = 0.13

$$I_{IL-13} = I_0\{\alpha e^{-t/\tau_{fast}} + (1-\alpha)e^{-t/\tau_{slow}}\}(1 - e^{-t/\tau_d})$$
$$I_{cross\ to\ IL-5} \approx 0$$

I_0: IL − 13 *maximum intensity*, α: ratio between faster and slower decay = 0.69
τ_{fast}: time constant of faster decay=0.36 [h], τ_{slow}: time constant of slower decay=7.8 × 10 [h]
τ_d: time constant of staining = 0.28 [h]

Figure S7: Standard curve of fluorescent immunoassay signals of recombinant human IL-5 or IL-13 sampled by micro-injecting proteins, Related to STAR Methods

(A) Time-resolved fluorescence immunoassay for recombinant human IL-5 microinjected into nanoliter wells in a TIRF-dish at different amounts performed with 22.5 nM of anti-mouse IL-5 detection antibody every 20 min. (B) Time-resolved fluorescence immunoassay for recombinant human IL-5 microinjected into nanoliter wells in a TIRF-dish at different amounts performed with 90 nM of anti-human IL-13 detection antibody every 20 min. The curves of the formula for $I_{IL\text{-}5}$ and $I_{cross\ to\ IL\text{-}13}$ fitted well with the data shown in panels a and b, respectively. (C) Kinetics of cross-reactivity of human IL-13 protein to human IL-5 fluorescence immunoassay was measured by depositing trace amounts of recombinant human IL-13 into a TIRF-dish carrying an immunoassay constructed with the human IL-5 antibody set. Each dot indicates the maximum intensity estimated by the formulas for $I_{IL\text{-}5}$ and $I_{cross\ to\ IL\text{-}13}$. The human anti-IL-13 antibody had cross-reactivity with the IL-5 sandwich immunocomplex, whereas the anti-IL-5 antibody had no cross-reactivity with the IL-13 sandwich immunocomplex. (D) Time-resolved fluoroimmunoassay for recombinant human IL-13 microinjected into nanoliter wells in a TIRF-dish at different amounts was observed with 90 nM of anti-human IL-13 detection antibody every 20 min. The fitted parameters were used in Fig. 4A, B.

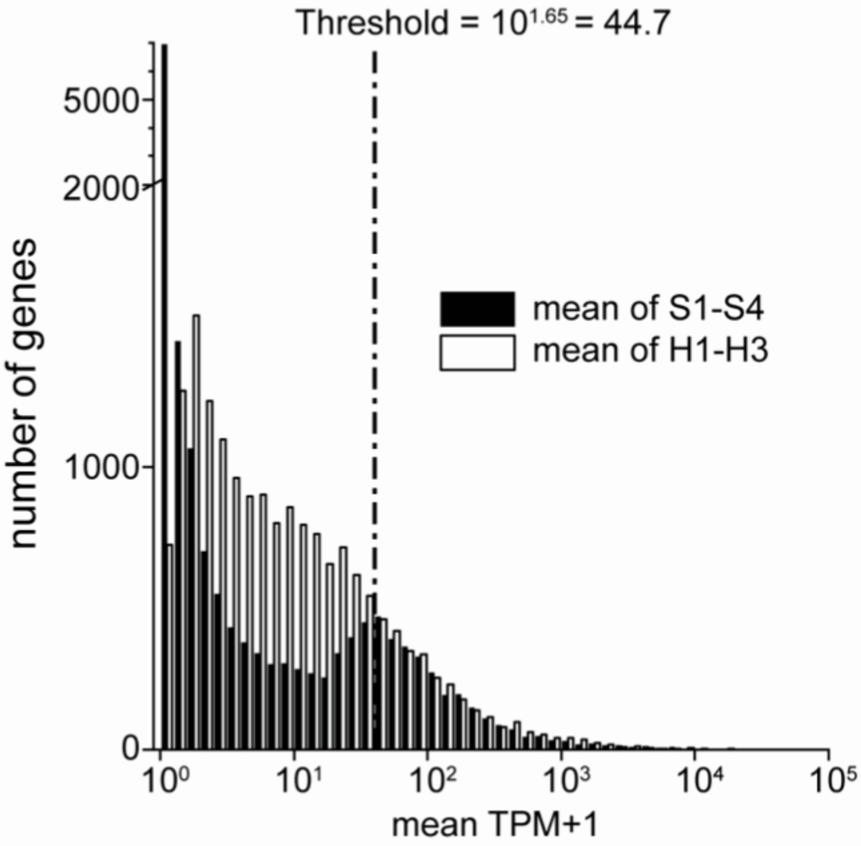

Figure S8: Distribution of transcripts per kilobase million (TPM) for retrieved hILC2s, Related to STAR Methods

Mean TPM of hyperactive or silent hILC2s. The values were increased by 1 to show all genes in a logarithmic manner. Silent hILC2s (S1 to S4), whose cDNA library was derived from a single cell, showed a cut-off pattern compared to multi-proliferated hyperactive hILC2s. We defined the confidence level associated with stochastically missing gene expression as TPM = 43.7. We assumed that the difference in gene expression was more reliable if the expression level of the gene exceeded this threshold, especially in silent hILC2s.
